# Revealing potential signaling pathways and hub genes related to monocytes in sepsis survivors and non-survivors based on single-cell RNA-seq and bulk RNA-seq data

**DOI:** 10.1590/1414-431X2025e14930

**Published:** 2025-10-17

**Authors:** Yanni Liu, Na Liu

**Affiliations:** 1 Department of Critical Care Medicine, Shandong Provincial Third Hospital, Cheeloo College of Medicine, Shandong University, Tianqiao District, Jinan City, Shandong Province, China

**Keywords:** Sepsis, Survivor, Non-survivor, Cell communication, Monocytes, Hub gene

## Abstract

Sepsis is a life-threatening organ dysfunction with a high incidence rate and mortality. The aim of this study was to investigate monocyte-related signaling pathways and hub genes in sepsis survivors and non-survivors. Sepsis-related data were downloaded from Gene Expression Omnibus (GEO) database. Cell annotation and cell communication analysis were performed to identify signaling pathways and ligand-receptor pairs related to monocytes. Immune cell infiltration, functional annotation, differential expression, and correlation analysis were performed to screen for hub genes associated with monocytes. In addition, survival analysis, transcription factors, and drug prediction were also performed on the hub genes. Compared with sepsis survivors, monocytes decreased in sepsis non-survivors. Cell communication results showed that monocytes were also the main signal transmitters and receivers in both the sepsis survivor and the sepsis non-survivor groups. A total of 25 signaling pathways related to monocytes were identified, such as MIF, ANNEXIN, GALECTIN, THBS, ITGB2, CCL, MHC-I, MHC-II, CD23, ICAM, and SEMA4. Subsequently, 6 hub genes (*CCR1*, *CD4*, *CD47*, *ITGAX*, *LILRB1,* and *PLXNB2*) associated with monocytes were identified. Univariate Cox analysis showed that *CD4*, *ITGAX*, *LILRB1*, *PLXNB2*, and age were associated with the prognosis of sepsis. Multivariate Cox analysis showed that ITGAX and age might be independent prognostic factors for sepsis. *ITGAX* and *CD4* are associated with transcription factors SPI1 and MYB, respectively. Moreover, drug prediction results showed that tregalizumab was an agonist of CD4. This study revealed the monocyte-associated signaling pathways and hub genes, which may contribute to the understanding of the molecular mechanisms of sepsis survivors and non-survivors.

## Introduction

Sepsis is a life-threatening organ dysfunction caused a dysfunctional response to infection by the host (1). With high incidence rate and mortality, sepsis has brought huge economic burden to the world. At the cellular and molecular level, the pathogenesis of sepsis is extremely complex, including the imbalance of inflammatory response, immune dysfunction, and other pathophysiological processes, which eventually lead to organ dysfunction (2).

Monocytes are mononuclear myeloid cells that develop in the bone marrow and circulate in the blood, which play an important role in both inflammation and homeostasis (3). The host immune response in sepsis is caused by leukocytes recognizing pathogens and danger signals. As a crucial component of leukocytes, monocytes play a significant role in defending against infections during sepsis (4). Monocyte count is associated with mortality in patients with sepsis, with decreased monocyte count in non-survivors of sepsis (5). In addition, previous studies have also found that a variety of molecules such as CD64, HMGB1, and TREM-1 mediate monocyte activation or recruitment, thereby affecting different clinical outcomes of sepsis. Therefore, it is essential to continue to understand the potential molecular regulatory mechanisms of monocytes and the associated genes in sepsis.

ScRNA-seq technology allows the dissection of gene expression at single-cell resolution (6). CellChat is a tool that enables quantitative inference, visualization, and analysis of intercellular communication networks to identify key features of intercellular communication in a given scRNA-seq dataset. In this study, we downloaded RNA-seq and scRNA-seq data for sepsis survivors and non-survivors from the Gene Expression Omnibus (GEO) database (http://www.ncbi.nlm.nih.gov/geo/). Subsequently, hub genes and signaling pathways associated with monocytes were identified by cell communication, differential expression analysis, functional annotation and other bioinformatics methods to reveal the potential molecular mechanisms involved in different clinical outcomes of sepsis. The aim of this study was to investigate monocyte-related signaling pathways and hub genes in sepsis survivors and non-survivors.

## Material and Methods

### Data sources

Datasets with sepsis gene expression data and complete clinical annotations were searched in the GEO database. Studies at the cellular or animal level and single sample were excluded. Studies with samples from child patients were also excluded. The sample type was blood and included blood collected within 24 h of admission. Moreover, the selected dataset should also include the survival and death outcomes. Finally, GSE167363, GSE95233, and GSE54514 datasets were included in this study (Supplementary Table S1). All data were downloaded on March 20, 2023.

Blood sample data from 6 sepsis survivors and 4 sepsis non-survivors of GSE167363 dataset were included in this study for subsequent cell analysis. Blood sample data from 22 healthy controls on day 1, 34 sepsis survivors on day 1, and 17 sepsis non-survivors on day 1 of GSE95233 dataset were included in this study as the experimental set for subsequent studies. Blood sample data from 18 healthy controls on day 1, 26 sepsis survivors on day 1, and 9 sepsis non-survivors on day 1 of GSE54514 dataset were included in this study as the validation set for subsequent studies. The GSE95233 dataset was designated as the experimental set mainly based on its relatively large sample size and the data from after 2016 (the sepsis 3.0 diagnostic criteria was released in 2016 (1)).

### Identification and annotation of cells

The Seurat (version 4.0.2) and SingleR (version 1.4.1) packages were used for analysis of scRNA-seq data in GSE167363. After merging 6 samples from sepsis survivors, a total of 28291 cells were included. Similarly, 4 samples from sepsis non-survivors were merged, and a total of 17492 cells were included. Quality control was first performed to exclude genes detected in <3 cells, cells with a total number of detected genes <200, and cells with mitochondrial expression genes ≥5%. Then, NormalizeData (object=scRNA, normalization. method=“LogNormalize”, scale factor=10000) normalization was used to screen for the top 1500 highly variable genes. In addition, the ScaleData (version 0.2.2) function was used to perform Z-score normalization of the data, and based on this, the principal component (PC) dimension reduction was performed to select the top 20 PCs. Subsequently, the t-distributed stochastic neighbor embedding (tSNE) algorithm was used to reduce the dimensionality of 20 initial PCs and perform cluster analysis in all cells. The resolution parameter was set to 0.5. Cluster analysis was performed by FindNeighbors and FindClusters. Different cell clusters have different gene expression profiles, and the set of marker genes for each cell cluster was obtained based on the screening criteria P<0.05 and |log_2_fold change (FC)|>1. The singleR package was used to identify and annotate different cell clusters based on the expression patterns of the marker genes.

### Analysis of immune cell infiltration

Gene sets that labeled the types of infiltrating immune cells in immune microenvironment were obtained from the study of Charoentong et al. (7). The GSVA package (version 1.38.2) was used for single-sample Gene Set Enrichment Analysis (ssGSEA) analysis. The enrichment score calculated by ssGSEA analysis was used to represent the relative abundance of immune cell infiltration in each sample. The Wilcoxon test was used to compare differences of immune cell infiltration between sepsis survivors and non-survivors. In addition, Spearman correlation analysis was also performed.

### Cell communication analysis

CellChat is a tool that enables quantitative inference, visualization, and analysis of intercellular communication networks. CellChat (version 1.5.0) can identify key features of intercellular communication in a given scRNA-seq dataset (GSE167363) and predict the putative function of signaling pathways that are currently poorly understood. CellChat uses network analysis and pattern recognition methods to predict the main signal inputs and outputs of cells, as well as how these cells and signals coordinate functions. In this study, CellChat was used to explore the connections between monocytes and other cells, and their changes in sepsis survivors and non-survivors were compared to obtain key ligand-receptor pairs. CellChat infers cell-cell communication based on gene expression data of cells (8). When the number of cells is too small, it may not accurately reflect the true gene expression characteristics and intercellular communication patterns of the cell type. Therefore, the cell types with cell number less than 200 were excluded in cell communication analysis.

### Function annotation

Gene Ontology (GO) is divided into three main categories, namely molecular functions (MF), cellular component (CC), and biological process (BP). GO functional enrichment analysis was performed using the GeneCodis 4.0 database (https://genecodis.genyo.es/). False discovery rate (FDR)<0.05 was the screening criterion.

### Identification of hub genes

Differential expression analysis was performed on the obtained ligand and receptor genes based on the GSE95233 dataset. Then, the correlation between significant differentially expressed genes and monocytes was analyzed (P<0.05). The differentially expressed genes associated with monocytes were considered hub genes. Subsequently, the hub genes were input into the STRING database (https://cn.string-db.org/) to construct a protein-protein interaction (PPI) network to study the regulatory relationship among genes.

Furthermore, sepsis survival information was downloaded from the GSE65682 dataset in the GEO database. Subsequently, consistent clustering was performed using the “ConsensusClusterPlus” package based on the expression values of hub genes in sepsis patients. Kaplan-Meier analysis was used to compare the survival differences among different subgroups. In addition, univariate and multivariate Cox analyses were used to determine potential prognostic factors. The clinical information statistics of the samples in the GSE65682 dataset are shown in Supplementary Table S2.

### Prediction of hub genes-related transcription factors (TFs) and drugs

The TFs related to hub genes were screened based on the TRRUST database (https://www.grnpedia.org/trrust/). Moreover, the differential expression of TFs between sepsis survivors and non-survivors was analyzed. Drugs related to hub genes were screened based on the DGIdb database (https://dgidb.org/).

### Real time PCR expression verification

A total of 18 blood samples from 14 sepsis survivors and 4 sepsis non-survivors were collected for real time PCR validation of hub genes. The main reagents required included blood RNA extraction kit (Magen, R4163-02, China), FastQuant cDNA first strand synthesis kit (TIANGEN, KR106, China), and SuperReal PreMix Plus (SYBR Green) (TIANGEN, FP205). Each experiment was repeated three times. GAPDH and ACTB were internal references. The 2^-△△CT^ method was used for relative quantitative analysis.

## Results

### Identification and annotation of monocytes

The 8 cell types annotated (Supplementary Figure S1) in the sepsis survivor group were B-cells (575), CD4+T-cells (2553), CD8+T-cells (166), dendritic cells (DC) (101), monocytes (2662), neutrophils (385), natural killer (NK) cells (905), and platelets (237). The 7 cell types annotated (Supplementary Figure S2) in the sepsis non-survivor were B-cells (517), CD4+T-cells (2386), hematopoietic stem cells (HSC) (57), monocytes (885), neutrophils (899), NK cells (303), and platelets (2170). The numbers in parentheses represent the number of cells for each cell type. B-cells, CD4+T-cells, monocytes, neutrophils, NK cells, and platelets were annotated in both sepsis survivor and non-survivor groups. The content of B-cells and CD4+T-cells did not change significantly between the sepsis survivor and non-survivor groups. Compared with the sepsis survivor group, monocytes and NK cells decreased in the sepsis non-survivor group, while neutrophils and platelets increased in the sepsis non-survivor group.

### Immune cell infiltration

**Figure 1:**
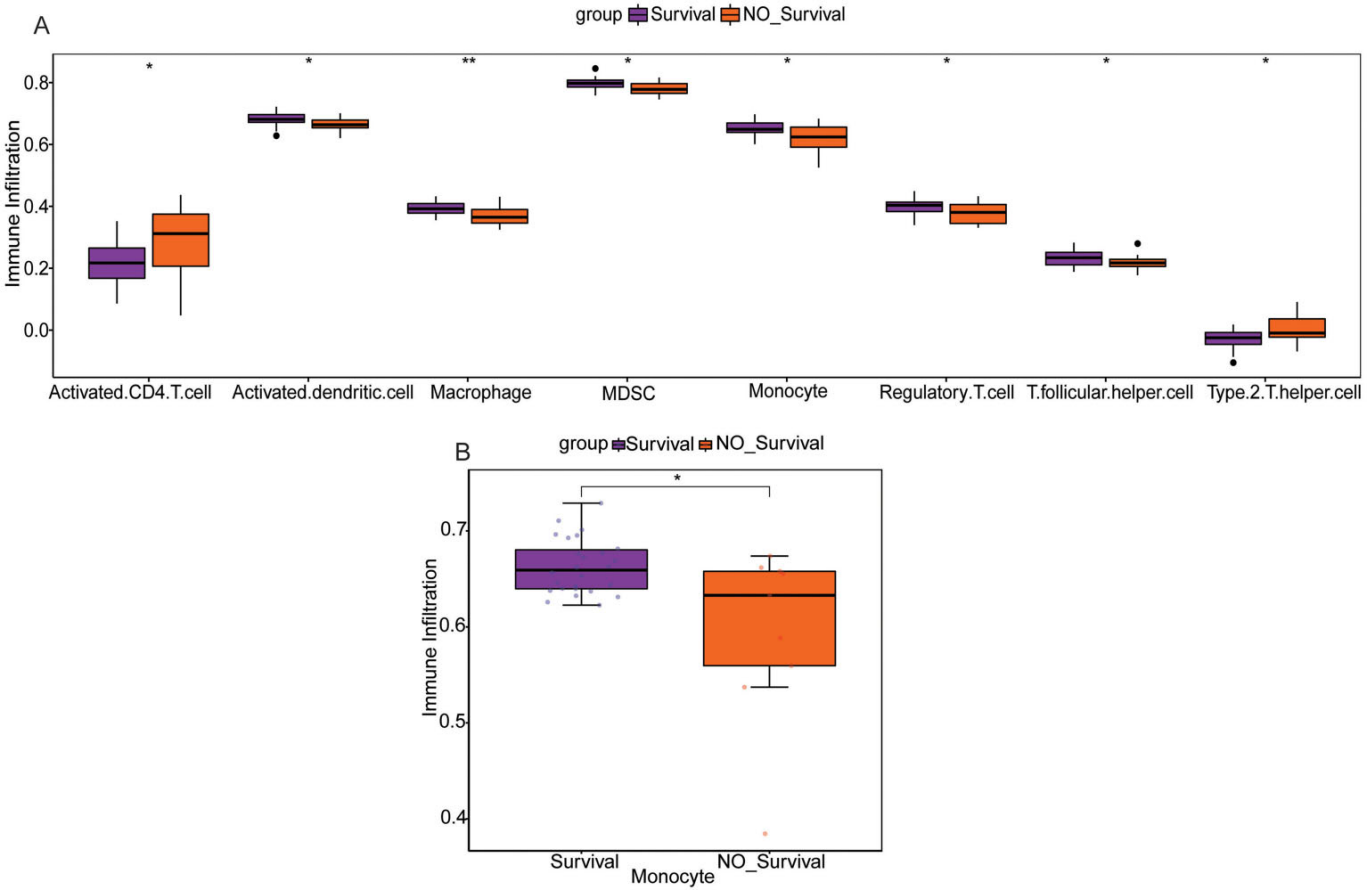
Immune cell infiltration. **A**, Significantly different immune cell infiltration between the sepsis survivor and non-survivor groups based on the GSE95233 dataset. **B**, Verification of monocyte infiltration status in the sepsis survivor and non-survivor groups based on GSE54514 datasets. Data are reported as median and interquartile range. *P<0.05; **P<0.01 (Wilcoxon test).

The ssGSEA algorithm was used to evaluate the relative abundance of immune cell infiltration. The results showed that among the 7 immune cell types annotated above, monocytes and activated dendritic cells were significantly reduced in the sepsis non-survivor group, while activated CD4+T cells were significantly increased in the sepsis non-survivor group (Figure 1A). In addition, the infiltration status of 7 annotated immune cells was validated in the GSE54514 dataset, and the results showed that only the infiltration levels of monocytes were significantly reduced in the sepsis non-survivor group (Figure 1B).

### Cell communication

**Figure 2:**
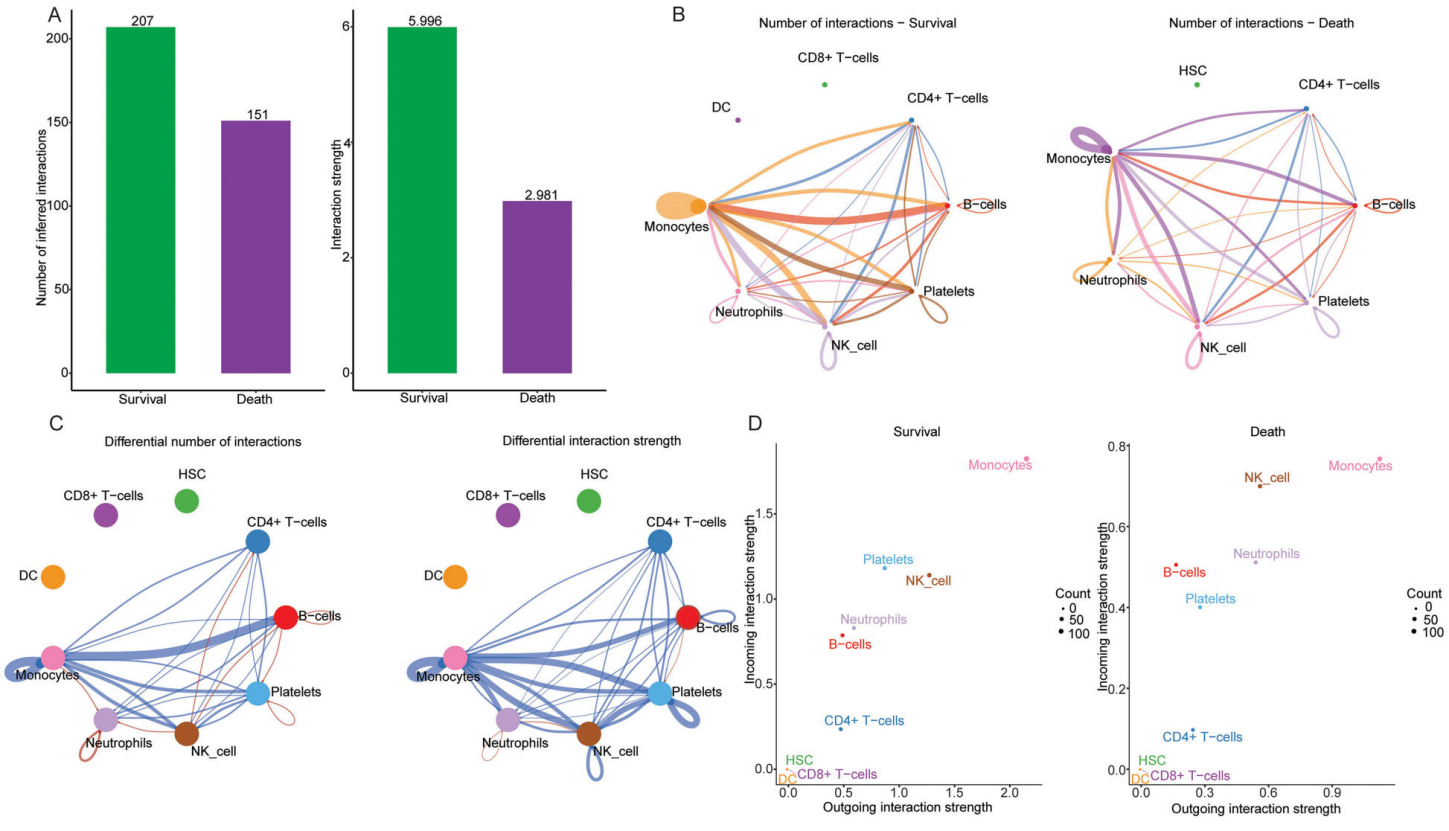
Comparison of cell communication between sepsis survivors and non-survivors. **A**, Comparison of the total number (left) and strength (right) of interactions between sepsis survivors and non-survivors. **B**, The number of interactions between any two cell types in the sepsis survivors and non-survivors. The thicker the line, the greater the number of interactions detected between different cell types. **C**, Differences in the number or strength of interactions between different cell populations in the sepsis survivors and non-survivors. **D**, The main sources and targets of signals in 2D space of sepsis survivors and non-survivors. Monocytes were the main signal transmitters (outgoing) and receivers (incoming) in both the sepsis survivor and the sepsis non-survivor groups.

The cell communication analysis for DC cells, CD8+T-cells, and HSC were not performed because the number of cells was less than 200. In the sepsis survivor group, a total of 59 ligand-receptor pairs were detected in the remaining 6 cell types, which were further divided into 27 signaling pathways. In the sepsis non-survivor group, a total of 28 ligand-receptor pairs were detected in the remaining 6 cell types, which were further divided into 16 signaling pathways. CellChat was used to compare the total number and strength of interactions between sepsis survivor and non-survivor groups. Cell communication was found to be more frequent in the sepsis survivor group (Figure 2A-C). From the perspective of the regulation between each cell, it is mainly manifested in the regulation between monocytes and B-cells. Subsequently, the outgoing signal interaction strength (outgoing interaction strength) and the incoming signal interaction strength (incoming interaction strength) of each cell in the 2D space were compared. Monocytes were the main signal transmitters (outgoing) and receivers (incoming) in both the sepsis survivor and the sepsis non-survivor groups (Figure 2D).

**Figure 3:**
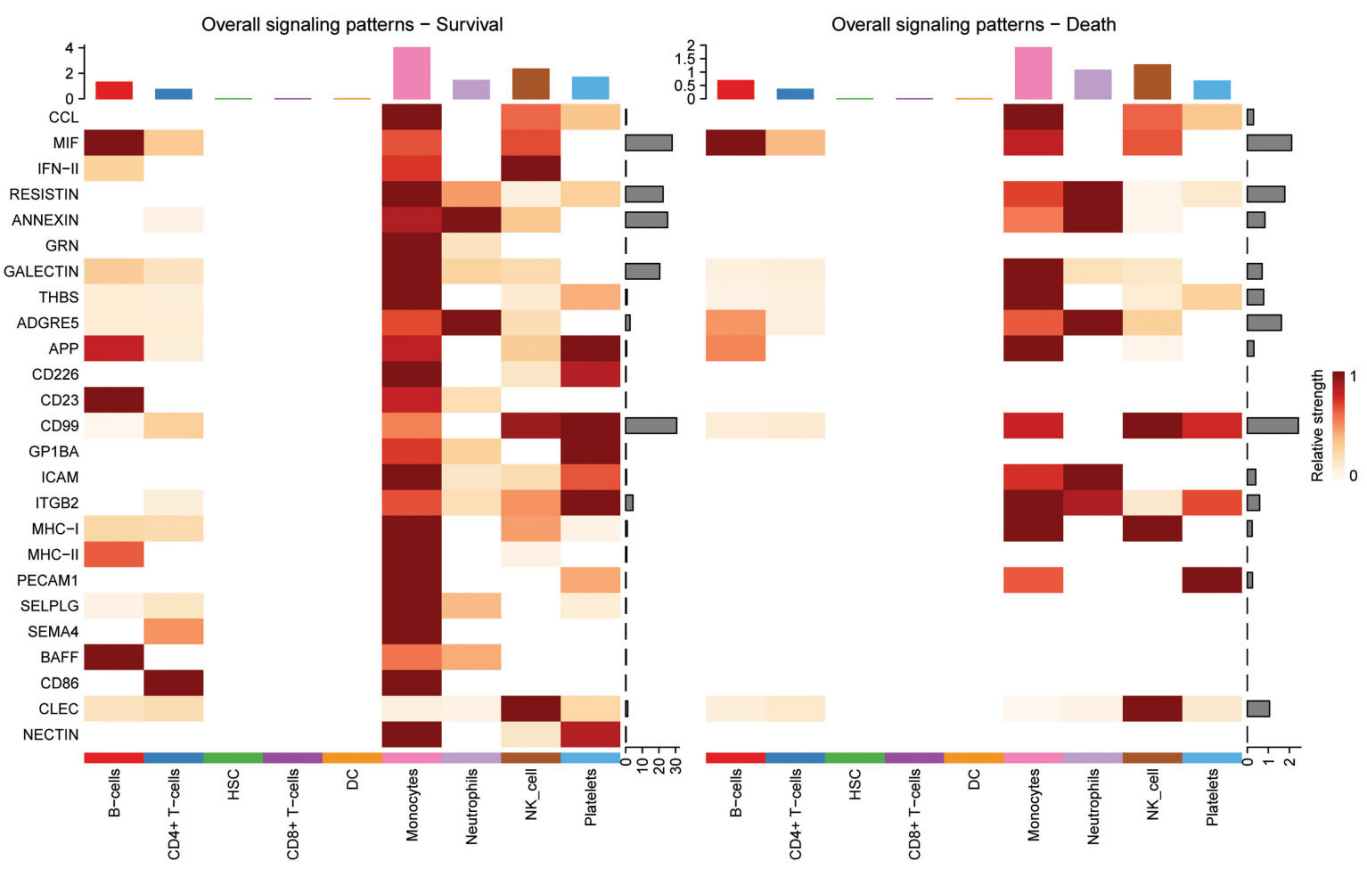
Comparison of the overall signals related to monocytes in the sepsis survivor and non-survivor groups side by side. The color bar represents the relative signaling strength of a signaling pathway. The top colored bar plot shows the total signaling strength of a cell group by summarizing all signaling pathways displayed in the heatmap. The right grey bar plot shows the total signaling strength of a signaling pathway by summarizing all cell groups displayed in the heatmap.

In addition, the information flow/interaction strength of each signaling pathway was also compared via CellChat (Supplementary Figure S3A). All identified signaling pathways related to monocytes from different groups were displayed, involving a total of 25 pathways (CCL, MIF, IFN-II, RESISTIN, ANNEXIN, GRN, GALECTIN, THBS, ADGRE5, APP, CD226, CD23, CD99, GP1BA, ICAM, ITGB2, MHC-I, MHC-II, PECAM1, SELPLG, SEMA4, BAFF, CD86, CLEC, and NECTIN) (Supplementary Figure S3B and C). Then, the outgoing and incoming signals were compared side by side, and showed that MIF, ANNEXIN, GALECTIN, THBS, and ITGB2 were the main outgoing signals in the monocytes of both groups, while CCL and MHC-I were the main incoming signals in the monocytes of both groups (Supplementary Figure S3D and E). Furthermore, a comparison of the overall signal profiles was conducted, and found that GALECTIN, THBS, CCL, and MHC-I were the major contributors to signal transmission in both groups of monocytes (Figure 3).

**Figure 4:**
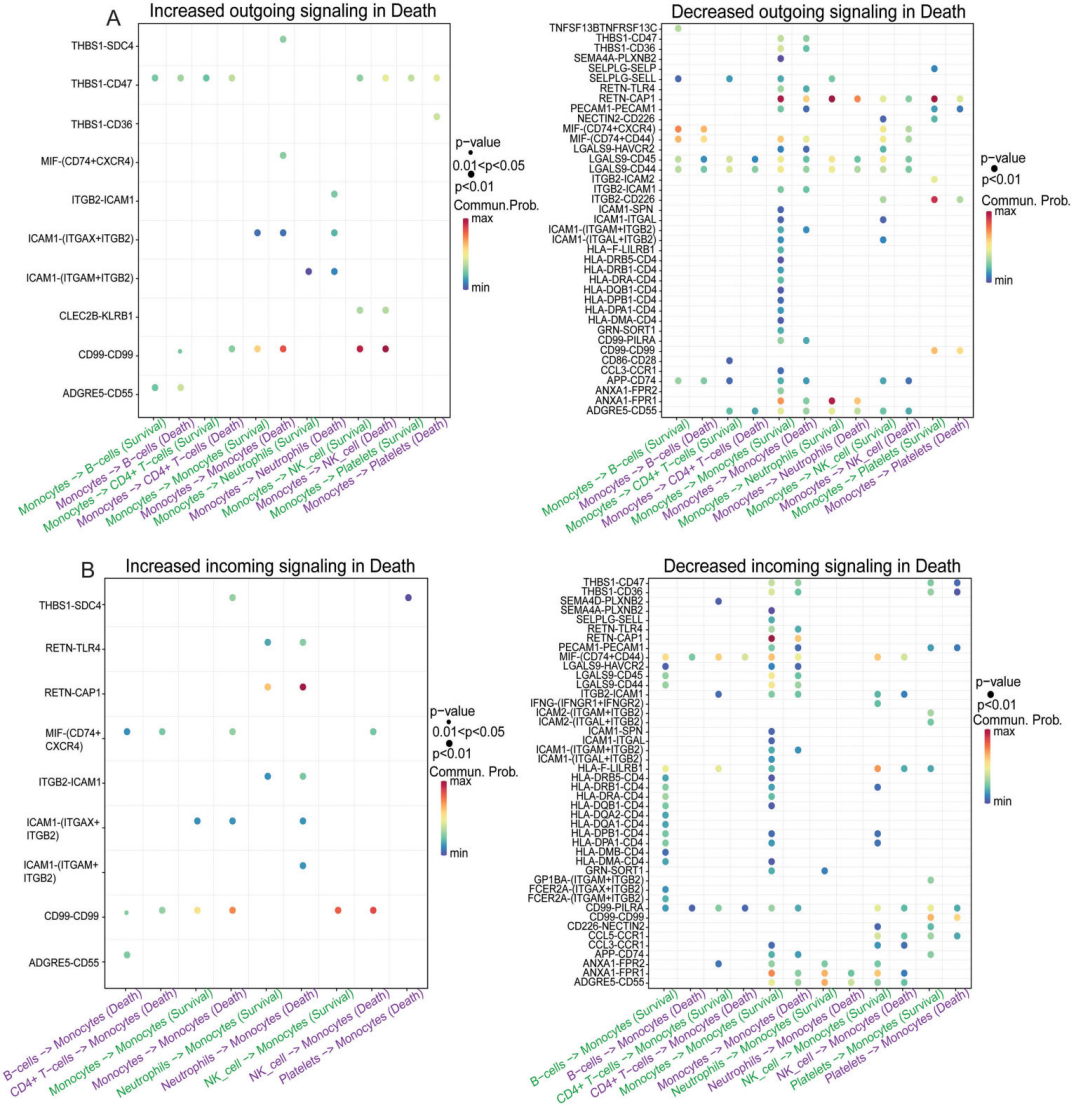
Identification of the up-regulated and down-regulated signaling ligand-receptor pairs of monocyte-associated signaling pathways based on communication probability comparison. **A**, Comparison of monocyte-associated outgoing communication probabilities mediated by ligand-receptor pairs. **B**, Comparison of monocyte-associated incoming communication probabilities mediated by ligand-receptor pairs. The vertical axis represents different ligand-receptor pairs, and the horizontal axis represents the cell communication between monocytes and other cells (“->” indicates the direction of communication between cells) and the sample state corresponding to the communication event (survival or death). Commun.Prob represents the communication probability, and the color from cold to warm indicates the communication probability from low to high.

**Figure 5:**
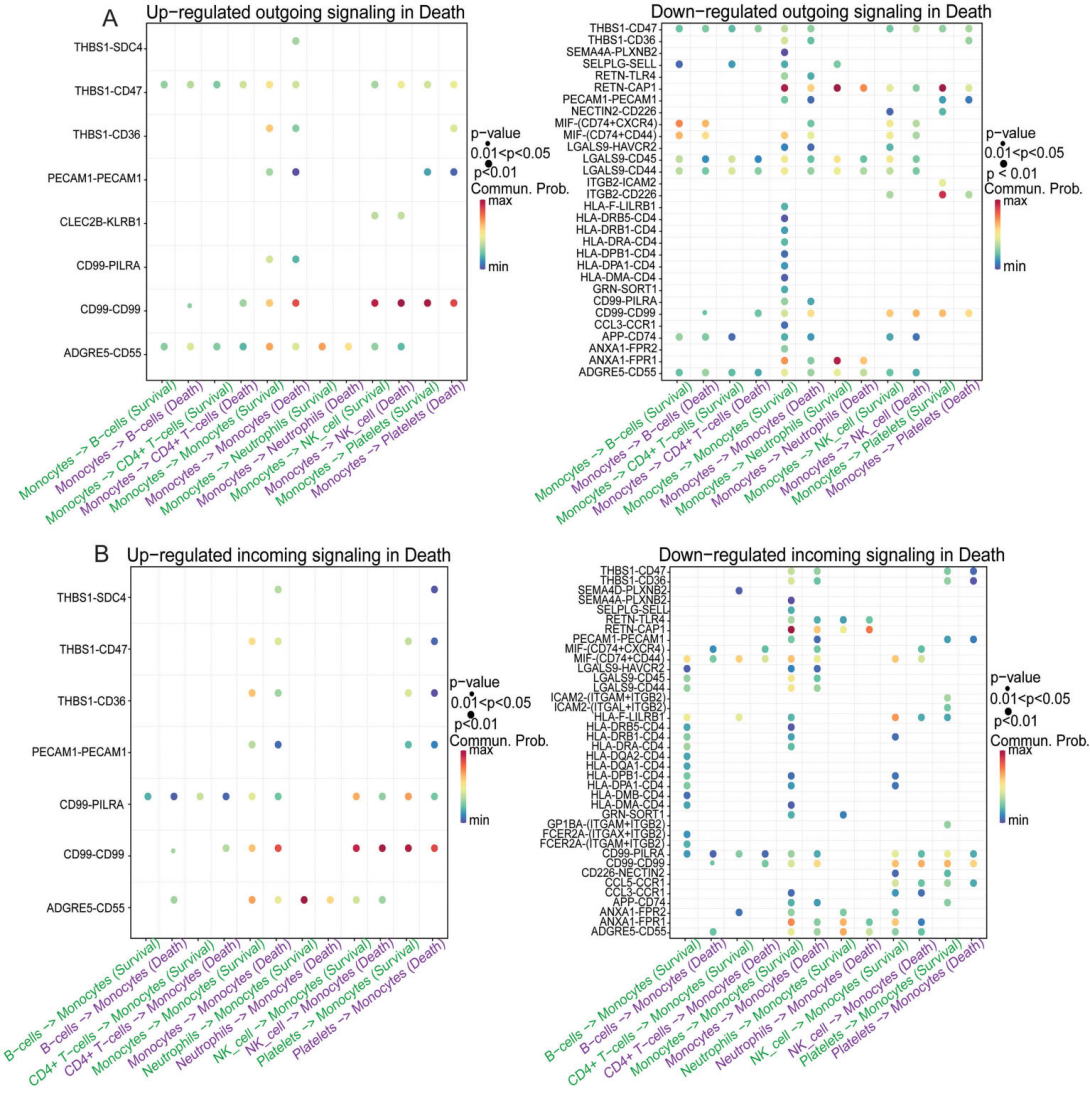
Identification of the up-regulated and down-regulated signaling ligand-receptor pairs of monocyte-associated signaling pathways based on differential gene expression analysis. **A**, Identification of up-regulated and down-regulated signaling ligand-receptor pairs in monocyte-associated outgoing signals based on differential gene expression analysis. **B**, Identification of up-regulated and down-regulated signaling ligand-receptor pairs in monocyte-associated incoming signals based on differential gene expression analysis. The vertical axis represents different ligand-receptor pairs, and the horizontal axis represents the cell communication between monocytes and other cells (“->” indicates the direction of communication between cells) and the sample state corresponding to the communication event (survival or death). Commun.Prob represents the communication probability, and the color from cold to warm indicates the communication probability from low to high.

Furthermore, the up-regulated and down-regulated signaling ligand-receptor pairs of monocyte-associated signaling pathways were also identified. CellChat provides two methods: one is to compare the communication probabilities between certain cell populations and other cell populations mediated by ligand-receptor pairs (Figure 4A and B), and the other is to identify up-regulated and down-regulated signaling ligand-receptor pairs based on differential gene expression analysis (Figure 5A and B). Ligand-receptor pairs such as ADGRE5-CD55, CD99-CD99, and CLEC2B-KLRB1 were shown to increase in outgoing signaling from monocytes in the non-survivor group in both methods, while SEMA4A-PLXNB2, HLA-F-LILRB1, and APP-CD74 were shown to decrease in outgoing signaling from monocytes in the non-surviving sepsis group in both methods. Ligand-receptor pairs such as ADGRE5-CD55, CD99-CD99, and THBS1-SDC4 were shown to increase in incoming signaling from monocytes in the non-survivor group in both methods, while HLA-DMB-CD4, ANXA1-FPR2, and FCER2A-(ITGAX+ITGB2) were shown to decrease in incoming signaling from monocytes in the non-surviving sepsis group in both methods. A total of 43 ligand-receptor pairs were identified, including 56 genes.

### Identification of hub genes associated with monocytes

**Figure 6:**
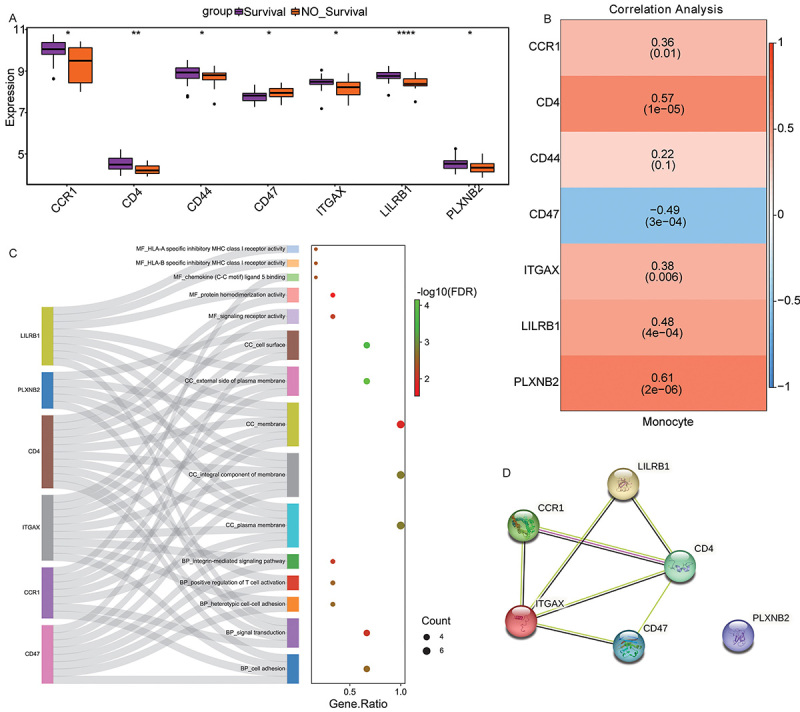
Identification of hub genes associated with monocytes. **A**, Box plot of differentially expressed genes. Data are reported as median and interquartile range. *P<0.05; **P<0.01; ****P<0.0001 (Wilcoxon test). **B**, Correlation between differentially expressed genes and monocytes. **C**, Gene Ontology (GO) functional enrichment analysis of hub genes. **D**, Protein-protein interaction (PPI) network of hub genes.

The 43 ligand-receptor pairs identified above contained a total of 56 genes. Among them, 7 of 56 genes were differentially expressed between sepsis survivor and sepsis non-survivor groups (P<0.05). Compared with the sepsis survivor group, CCR1 (log2FC=-0.0962, P=0.0276), CD4 (log2FC=-0.0971, P=0.0032), CD44 (log2FC=-0.0353, P=0.0460), ITGAX (log2FC=-0.0492, P=0.0341), LILRB1 (log2FC=-0.0621, P=1e−05), and PLXNB2 (log2FC=-0.0645, P=0.0397) were down-regulated in the sepsis non-survivor group, while CD47 (log2FC= 0.0355, P=0.0324) was up-regulated in the sepsis non-survivor group (Figure 6A). Correlation analysis showed that CCR1 (cor=0.36, P=0.01), CD4 (cor=0.57, P=1e−05), CD47 (cor=-0.49, P=3e−04), ITGAX (cor=0.38, P=0.006), LILRB1 (cor=0.48, P=4e−04), and PLXNB2 (cor=0.61, P=2e−06) were significantly correlated with monocytes, and the highest correlation between PLXNB2 and monocytes was 0.61 (Figure 6B). Subsequently, genes (CCR1, CD4, CD47, ITGAX, LILRB1, and PLXNB2) that were significantly differentially expressed (P<0.05) and significantly correlated with monocytes (P<0.05) were defined as hub genes.

The expressions of hub genes were verified in the GSE54514 dataset. The results showed that CD4, ITGAX, and PLXNB2 were down-regulated in the sepsis non-survivor group, while CD47 was up-regulated in the sepsis non-survivor group (Supplementary Figure S4), which was consistent with the expression results in the GSE95233 dataset. In addition, real time PCR was also performed based on 14 sepsis survivor blood samples and 4 sepsis non-survivor blood samples to verify the expression of CCR1, CD4, CD47, ITGAX, LILRB1, and PLXNB2. The results showed that CCR1, CD4, ITGAX, LILRB1, and PLXNB2 had a down-regulated trend in the sepsis non-survivor group, while CD47 had an up-regulated trend (Supplementary Figure S5). Real time PCR results showed that the expression trend of hub genes was consistent with bioinformatics analysis, but it was not significant. This may be due to the small sample size. The small sample size, particularly the limited number of non-survivors, was caused by the difficulty in recruiting patients who met our strict inclusion requirements within the limited research time. In the later period, samples will continue to be collected for further verification.

Cell communication analysis showed that CCR1, acting as a receptor, binds to CCL3 and CCL5 and participates in the CCL pathway. CD4, acting as a receptor, binds to HLA-DPA1, HLA-DPB1, HLA-DQA1, HLA-DMA, HLA-DMB, HLA-DQA2, HLA-DQB1, HLA-DRA, HLA-DRB1, and HLA-DRB5 and participates in the MHC-II pathway. CD47, acting as a receptor, binds to THBS1 and participates in the THBS pathway. ITGAX, acting as a receptor, binds to FCER2 and ICAM1 and participates in the CD23 and ICAM pathways, respectively. LILRB1, acting as a receptor, binds to HLA-F and participates in the MHC-I pathway. PLXNB2, acting as a receptor, binds to SEMA4A and SEMA4D and participates in the SEMA4 pathway.

GO functional analysis was performed on 6 hub genes. GO functional analysis found that in the GO:BP term, hub genes were mainly enriched in cell adhesion and signal transduction (Figure 6C). In the GO:CC term, hub genes were mainly enriched in membrane, integral component of membrane and plasma membrane (Figure 6C). In the GO:MF term, hub genes were mainly enriched in signaling receptor activity and protein homodimerization activity (Figure 6C). In addition, a PPI network was constructed, and the results showed that CD4 and ITGAX had the most interacting genes (Figure 6D). The interaction score (0.952) between CD4 and ITGAX was the highest (Supplementary Table S3).

**Figure 7:**
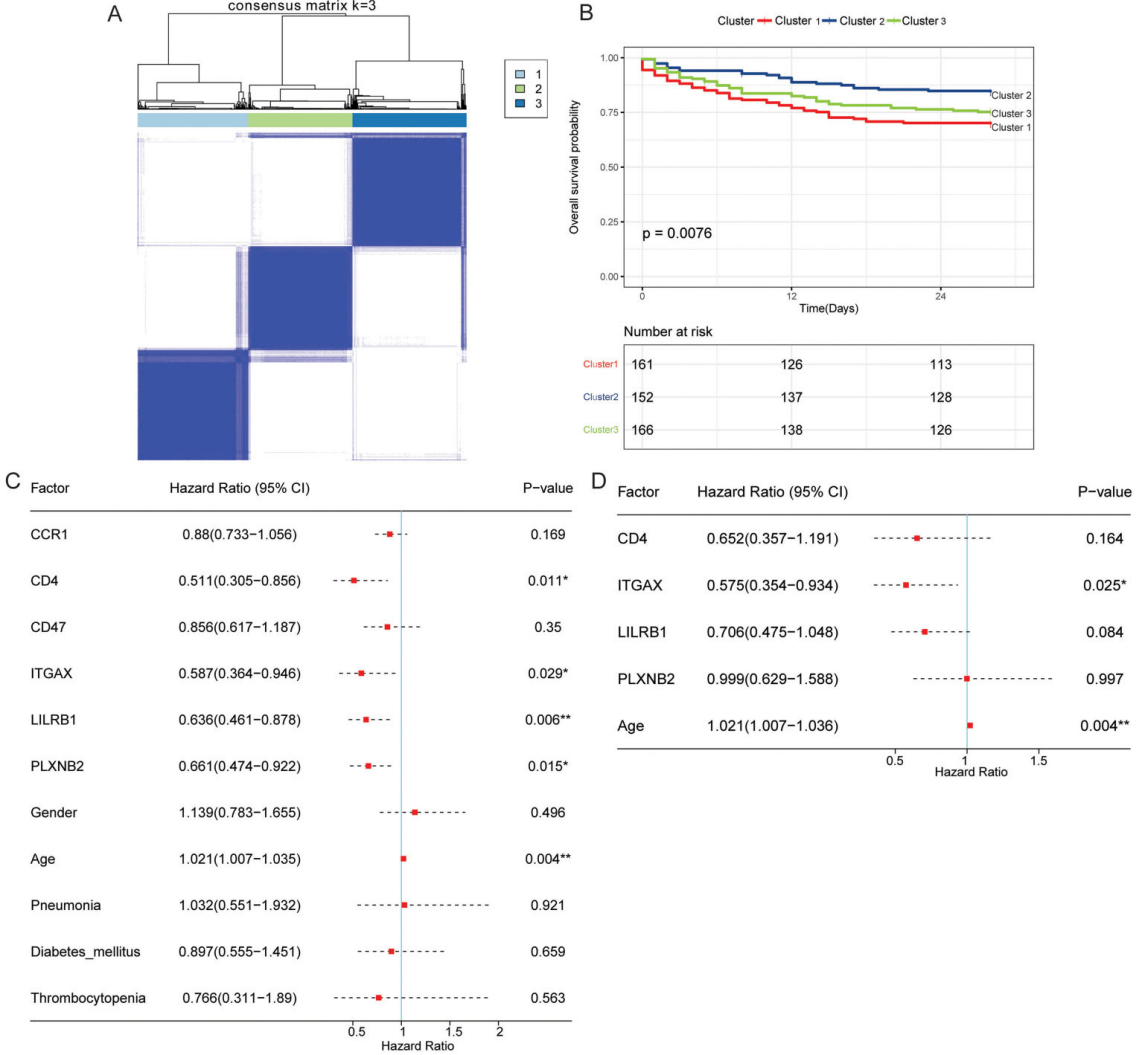
Consistent clustering and univariate and multivariate Cox analyses. **A**, Clustering result of hub genes expression profiles with k=3. **B**, Survival curve results of k=3. **C**, Univariate Cox analysis. **D**, Multivariate Cox analysis. *P<0.05; **P<0.01.

Based on the expression values of hub genes in the GSE65682 dataset, 3 subtypes (cluster 1, cluster 2, and cluster 3) with significant survival differences were determined after consensus clustering (Figure 7A). Among the 3 subtypes, the prognosis of patients in cluster 2 was significantly better than those of cluster 1 and cluster 3 (Figure 7B). Univariate Cox analysis showed that CD4, ITGAX, LILRB1, PLXNB2, and age were associated with the prognosis of sepsis (Figure 7C). Multivariate Cox analysis showed that ITGAX and age might be independent prognostic factors for sepsis (Figure 7D).

### Prediction of hub genes related TFs and drugs

**Figure 8:**
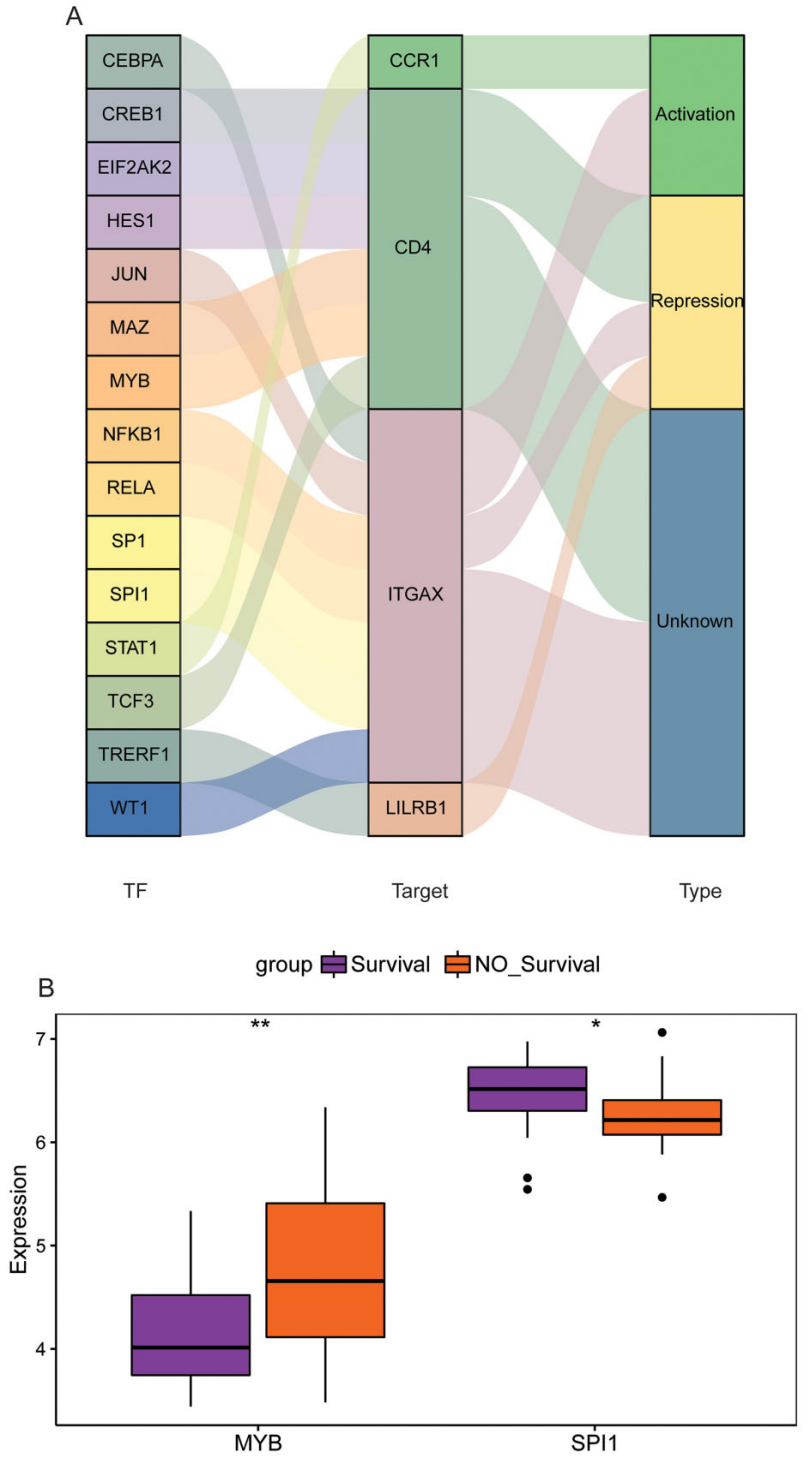
Prediction of hub genes-related transcription factors (TFs). **A**, Identification of hub genes-related TFs based on the TRRUST database. **B**, Significantly different TFs between sepsis survivor and non-survivor groups. Data are reported as median and interquartile range. *P<0.05; **P<0.01 (Wilcoxon test).

**Figure 9:**
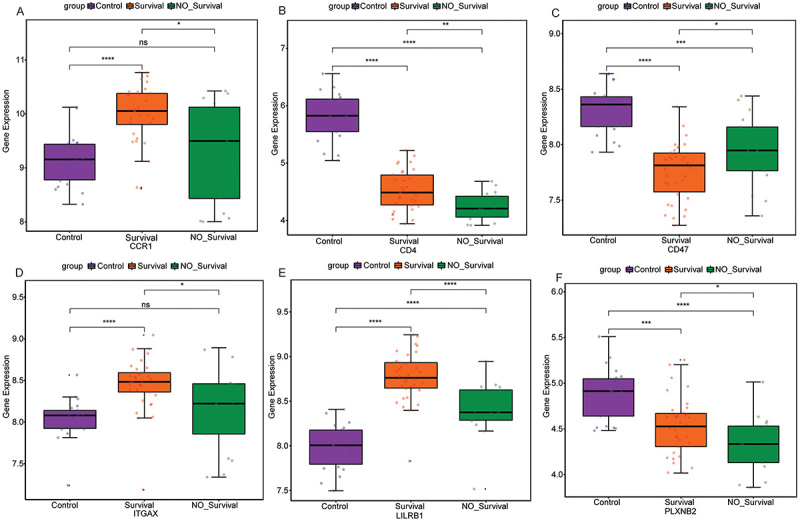
Differential expression of hub genes among the normal control group, sepsis survivor group, and sepsis non-survivor group based on GSE95233 data set. **A**, Differential expressions of CCR1; **B**, CD4; **C**, CD47; **D**, ITGAX; **E**, LILRB1; and **F**, PLXNB2. Data are reported as median and interquartile range. *P<0.05; **P<0.01; ***P<0.001; ****P<0.0001 (Wilcoxon test). ns: no statistical significance.

**Figure 10:**
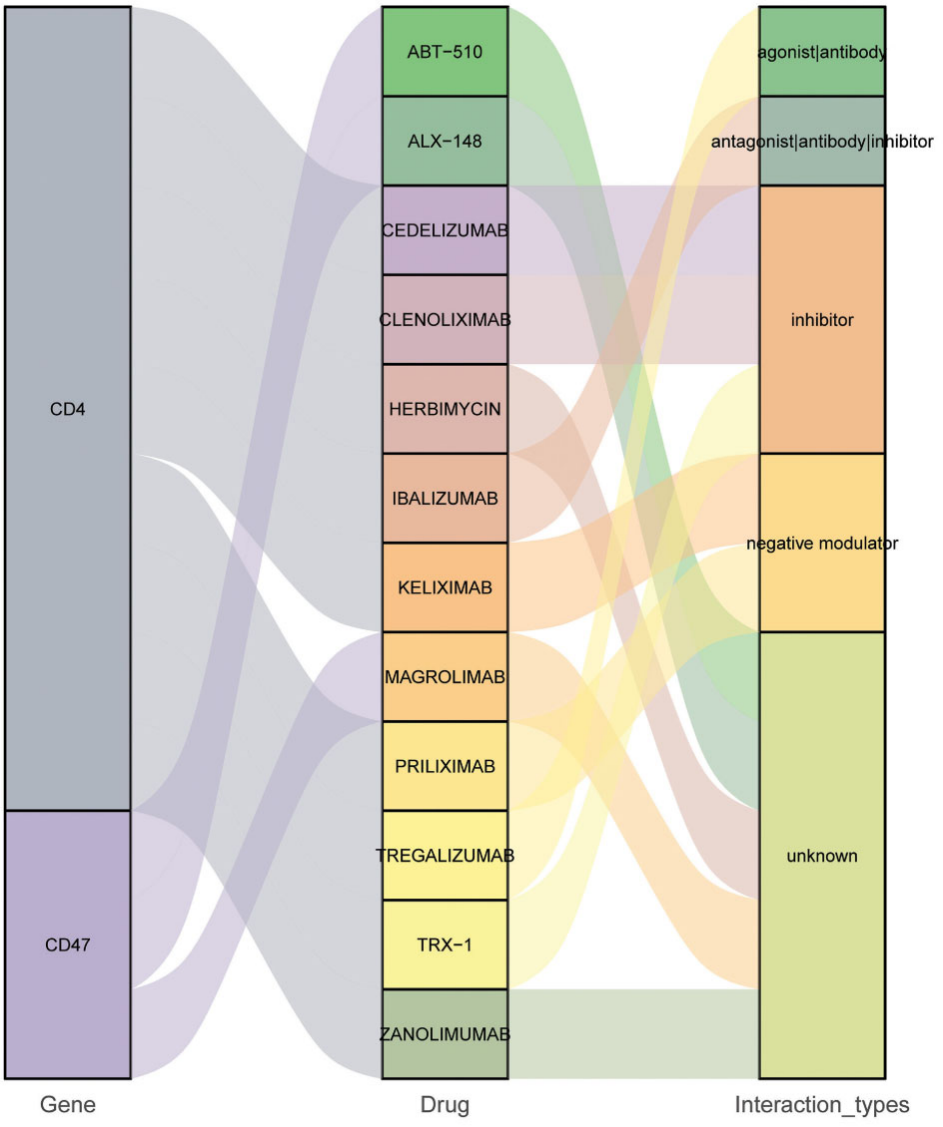
Related drugs for CD4 and CD47. The left column shows the name of the gene, the middle column shows the name of the drug, and the right column shows the type of action of the drug on the gene.

TFs associated with hub genes were identified based on the TRRUST database. A total of 15 TFs were identified, including 1 related to CCR1, 1 related to LILRB1, 6 related to CD4, and 7 related to ITGAX (Figure 8A). No CD47- and PLXNB2-related TFs were identified. The results of differential expression analysis in GSE95233 showed that MYB was significantly up-regulated in the sepsis non-survivor group, while SPI1 was significantly down-regulated in the sepsis non-survivor group (Figure 8B). The differential expression of hub genes among the normal control group, sepsis survivor group, and sepsis non-survivor group was also analyzed based on GSE95233 (Figure 9). Compared with the normal control group, CCR1, ITGAX, and LILRB1 were up-regulated in the sepsis survivor group, while CD4, CD47, and PLXNB2 were down-regulated. Compared with the normal control group, LILRB1 was up-regulated in the sepsis non-survivor group, while CD4, CD47, and PLXNB2 were down-regulated. Moreover, we also found that the expression of CD4 and PLXNB2 decreased sequentially in the normal control, sepsis survivor, and sepsis non-survivor groups. Subsequently, drugs associated with CD4, CD47, LILRB1, and PLXNB2 were predicted based on the DGIdb database. It should be noted that the drug predictions derived from DGIdb are exploratory in nature. These results are primarily intended to generate hypotheses for future research rather than to draw definitive conclusions about the therapeutic potential of these agents. The results showed that only CD4 and CD47 were predicted to be related to the relevant drugs (Figure 10). Tregalizumab is a CD4 agonist, which may have a certain effect on sepsis. However, it is crucial to emphasize that this remains a hypothesis that requires further experimental and clinical validation.

## Discussion

Monocytes, a type of leucocyte, are circulating myeloid cells and important innate effector cells involved in pathogen recognition and elimination. Sepsis is a life-threatening organ dysfunction with a high incidence rate and mortality. The abnormal activation and functional disorders of monocytes are key pathological features and their molecular regulatory networks remain unclear. In this study, multiple bioinformatics methods were used to integrate sepsis RNA-seq and scRNA-seq data, which can more accurately and comprehensively analyze the transcriptome characteristics of sepsis compared to previous studies. In addition, this study focuses on describing the unique role and value of monocytes in sepsis survivors and non-survivors. The identification of monocyte-related hub genes, ligand-receptor pairs, and signaling pathways lays a foundation for further study of the mechanism of monocytes in sepsis.

The cell communication analysis demonstrated that monocytes served as the primary signal transmitters and receivers in both the sepsis survivor and non-survivor groups. A total of 25 signaling pathways related to monocytes were identified. The outgoing and incoming signals were compared side by side, and the results showed that MIF, ANNEXIN, GALECTIN, THBS, and ITGB2 were the main outgoing signals in the monocytes of both groups, while CCL and MHC-I were the main incoming signals in the monocytes of both groups. Moreover, the overall signals were compared, and it was found that GALECTIN, THBS, CCL, and MHC-I were the major contributors to signal transmission in both groups of monocytes. Previous studies have shown that MIF (9), ANNEXIN (10), GALECTIN (11), THBS (12), ITGB2 (13), CCL (14), and MHC-I (15) signals are involved in the regulation of molecular mechanisms of sepsis. Subsequently, 6 hub genes (CCR1, CD4, CD47, ITGAX, LILRB1, and PLXNB2) were identified that may play an important role in regulating sepsis survival.

CCR1 is expressed by a wide range of leukocytes, including monocytes. The interaction of CCR1 and CCL5 has been demonstrated to exacerbate the innate immune response during sepsis (16). Herein, cell communication analysis showed that CCR1, as a receptor, binds to CCL3 and CCL5 ligands and participates in the CCL pathway. CCR1 may be involved in the molecular regulatory mechanism of sepsis by regulating the CCL pathway. ITGAX, also known as CD11C, is a member of leukocyte adhesion molecule β2 integrins. Its deficiency is associated with increased apoptosis and significant loss of hematopoietic stem and progenitor cells in sepsis and bone marrow transplantation (17). ITGAX is a key regulator of neonatal sepsis (13). ITGAX is critical for the regulation of neutrophil maturation, and activation of ITGAX could be a potential target for sepsis therapy (18). The expression of ITGAX in monocytes of patients with septic shock was decreased (19). Herein, ITGAX, as a receptor, binds to FCER2 and ICAM1 ligands and participates in the CD23 and ICAM pathways, respectively. CD23 may be involved in the regulation of sepsis death (20). ICAM is related to the survival rate of sepsis, and it also affects leukocyte infiltration and interstitial thickening in lung tissue (21). Moreover, ITGAX is also related to the TF SPI1. The expression of SPI1 is abnormal and mediates autophagy of peripheral blood monocytes in sepsis (22). SPI1 expression is also associated with sepsis survival (23). SPI1 can also positively regulate ITGAX expression (24). Until now, no relevant study on SPI1 regulating ITGAX in sepsis has been found, and this is the first report. Therefore, further investigation into the specific mechanisms of CCR1 and ITGAX in sepsis is warranted, as it may contribute to the development of more effective sepsis treatments.

The CD4 molecule performs multiple functions on a variety of cell types, including as adhesion molecules, inducers of cell activation, and chemotactic receptors. A previous study reported that CD4 is the hub gene in sepsis, and its expression is decreased in sepsis samples (25). CD4 expression of monocytes is significantly reduced in patients with septic shock and is associated with patient prognosis (26). In this study, it was shown that not only CD4 is involved in the MHC-II pathway, but also related to the TF MYB. The MHC-II pathway (27) and TF MYB (28) are both involved in regulating the mechanism of sepsis. Moreover, it has been reported that MYB plays an important role in CD4 expression (29). MYB can act as a transcription suppressor in CD4 silencers or as a transcription activator in CD4 promoters (30). However, the molecular regulatory mechanism of MYB on CD4 in sepsis remains to be further studied. CD47 is a transmembrane protein, expressed in different cell types, such as T cells, monocytes, and nerve cells, which belongs to the immunoglobulin superfamily (31). Moreover, it is also involved in the regulation of vascular function in sepsis (32). Herein, CD47, as a receptor, binds to THBS1 ligand and participates in the THBS pathway. In addition, drugs related to CD4 and CD47 were also queried based on the DGIdb database. Among them, tregalizumab is a CD4 agonist, which may have a certain effect on sepsis. In the sepsis non-survivor group, the expression of CD4 decreased, while the expression of CD47 increased. The results of this study combined with previous studies have shown that CD4 and CD47 play an important regulatory role in sepsis. Clinically, regulating the expression of CD4 and CD47 can help to improve the severity of sepsis.

LILRB1 is a receptor in leukocytes that plays a role in regulating immune responses (33). The expression of surface markers on human monocytes, including LILRB1, changes after stimulation with *Staphylococcus aureus* (34). PLXNB2, also known as PLEXB2, is involved in regulating the apoptosis process of monocytes (35). Previous studies have shown that PLXNB2 can regulate the proliferation and invasion of ovarian cancer (36,37). PLXNB2 participates in the inflammatory pathogenesis of psoriasis by binding to CD100 to activate NF-κB and the NLRP3 inflammasome (38). In this study, cell communication analysis showed that LILRB1 and PLXNB2 were involved in MHC-I and SEMA4 pathways as receptors, respectively. MHC-I is associated with the severity of sepsis (15,39). Semaphorins (SEMA) and their receptors play important roles in innate immune responses and acute inflammatory clinical diseases (40). Therefore, we speculate that LILRB1 and PLXNB2 may affect the progression of sepsis by modulating MHC-I and SEMA4 pathways. However, the specific molecular mechanism still needs to be further studied and verified.

It should be noted, however, that the study is not without limitations. Firstly, this study is a bioinformatics analysis based on public dataset data, which is a challenge in terms of data quality and standardization. Therefore, it is necessary to collect a large number of clinical samples for validation in the later stage. Secondly, in the cell communication analysis, DC cells, CD8+T-cells, and HSC were excluded, which may lead to incomplete understanding of the intercellular communication network in sepsis, and may miss some key immune regulatory links and signaling pathways. In the future, in-depth single-cell analyses of these cells will be needed. Thirdly, the specific mechanism of action between hub genes, monocytes, and signaling pathways still needs to be studied *in vitro*, including cell and animal model experiments. Fourthly, real time PCR results showed that the expression trend of hub genes was consistent with bioinformatics analysis, but it was not significant. This may be due to the small sample size. In the later period, samples will continue to be collected for further verification.

This study showed that monocytes were reduced in sepsis non-survivors compared to survivors and served as main signal transmitters and receivers in both groups, indicating they play a role in determining the survival status of sepsis patients. Moreover, 25 signaling pathways and 6 hub genes related to monocytes were identified. Abnormal expression of hub genes in the sepsis non-survivor group suggests that clinical regulation of their expression levels may contribute to sepsis treatment. In a word, the identified signaling pathways and hub genes related to monocytes in this study contribute to understanding the molecular mechanisms of sepsis. Moreover, this study has laid a theoretical foundation for future research.

## Supplementary Material

Click to view [pdf].
