# Supplementary material for: Revealing potential signaling pathways and hub genes related to monocytes in sepsis survivors and non-survivors based on single-cell RNA-seq and bulk RNA-seq data
Source: Braz J Med Biol Res. 2025 Oct 17;58:e14930. doi: 10.1590/1414-431X2025e14930 (PMC12534014; doi:10.1590/1414-431X2025e14930)
Supplement: Supplementary file 1 [file 1414-431X-bjmbr-58-e14930-suppl.pdf]

Figure S1. Single cell sequencing analysis and cell annotation in sepsis survivor group. **A**, Violin diagram shows the nFeature_RNA, nCount_RNA, and Percent.mt of each sequenced cell after quality control screening. **B**, Correlation analysis between Percent.mt and nCount_RNA, as well as nFeature_RNA and nCount_RNA. **C**: Top 1500 highly variable genes. **D**, Analysis of the top 20 principal components. **E**, Distribution of cells in each sample between PC1 and PC2. **F**, The tSNE projection of the cells. Each point corresponds to a separate cell. **G**, Annotation of 14 independent cell clusters into 8 cell types. **H**, Presentation of cell annotation results. **I**, Cell annotation diagnosis.

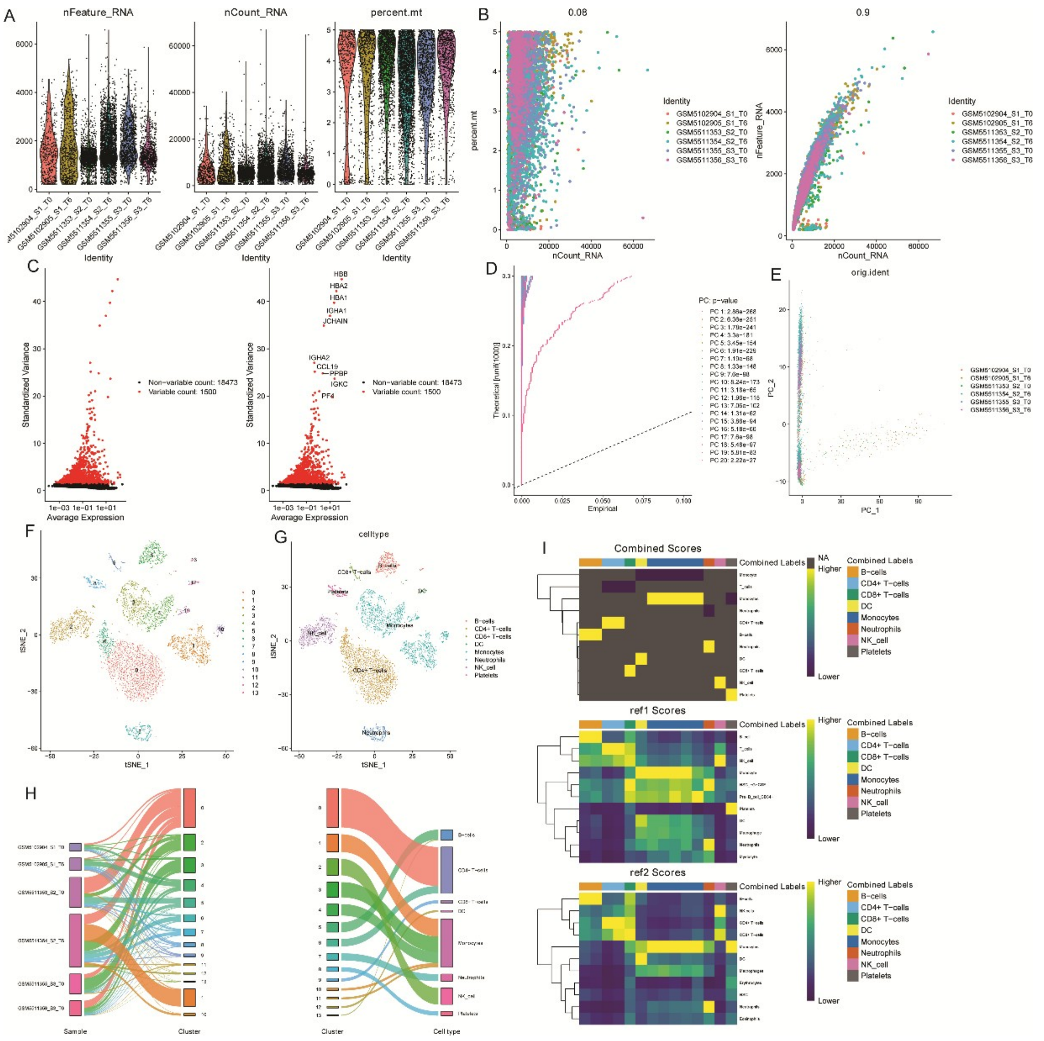

Figure description: In the sepsis survivor group, a total of 7,584 cells and 19,973 genes were identified according to quality control criteria that excluded low-quality cells and genes (1A). Correlation analysis found that nCount was not correlated with Percent.mt, but was highly correlated with nFeature (1B). The NormalizeData normalization process screened out the top 1500 highly variable genes (1C). The ScaleData function performs Z-score standardization on the data and then carries out PC dimension reduction to select the top 20 PC (1D). The distribution of cells in each sample between PC1 and PC2 is shown in Figure 1E. The tSNE algorithm divided the cells of the sepsis survivor group into 14 independent clusters (1F). Subsequently, 14 independent cell clusters were annotated into 8 types of cells through singleR package based on the expression pattern of the marker gene (1G). Clusters of 1, 3, 4, 10, and 11 were annotated as monocytes (1G and H). In addition, the annotation results were also diagnosed, and the results showed that monocytes had a higher score (1I).

Figure S2. Single cell sequencing analysis and cell annotation in sepsis non-survivor group. **A**, Violin diagram shows the nFeature_RNA, nCount_RNA and Percent.mt of each sequenced cell after quality control screening. **B**, Correlation analysis between Percent.mt and nCount_RNA, as well as nFeature_RNA and nCount_RNA. **C**, Top 1500 highly variable genes. **D**, Analysis of the top 20 principal components. **E**, The distribution of cells in each sample between PC1 and PC2. **F**, The tSNE projection of the cells. Each point corresponds to a separate cell. **G**, Annotation of the 13 independent cell clusters into 7 cell types. **H**, Presentation of cell annotation results. **I**, Cell annotation diagnosis.

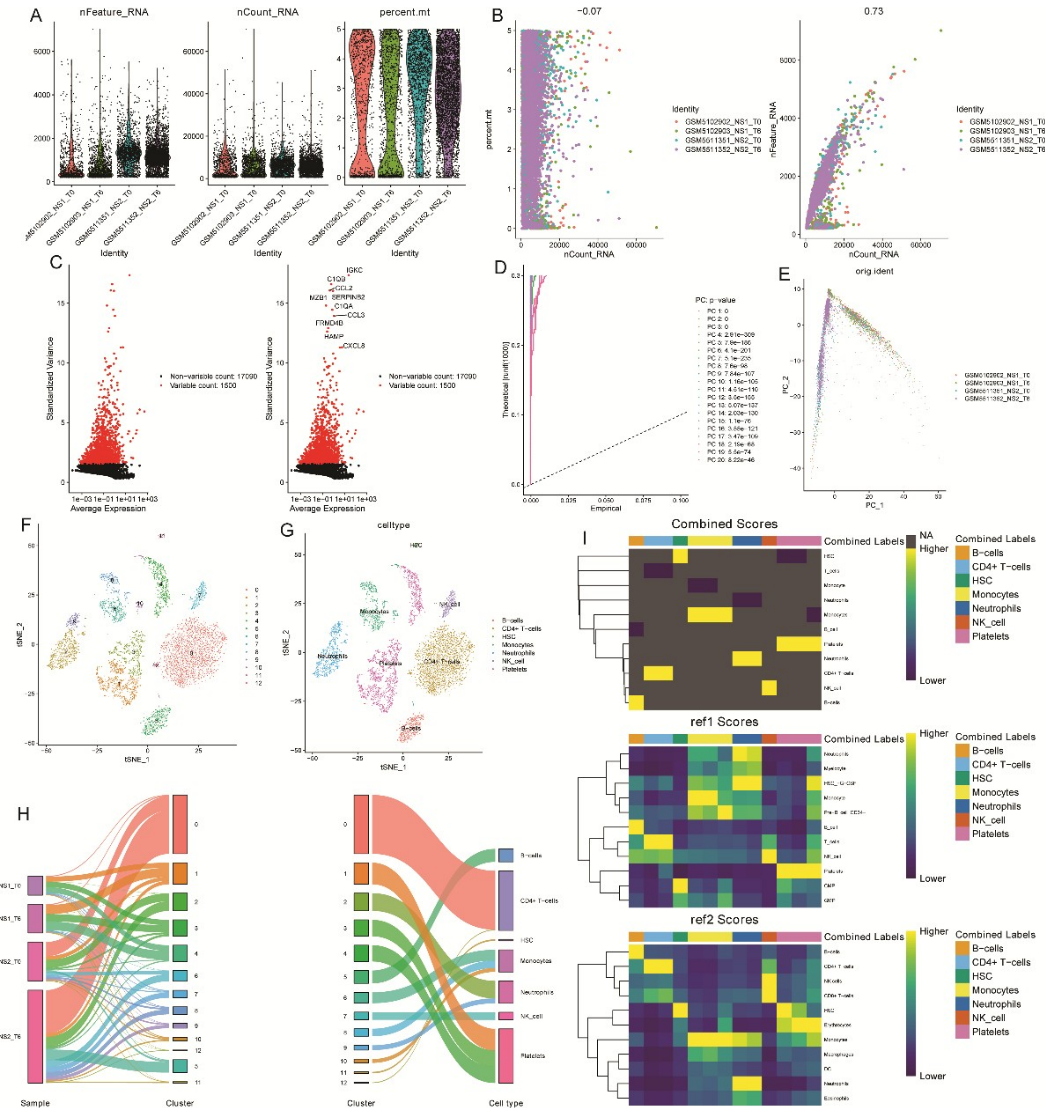

Figure description: In the sepsis non-survivor group, a total of 7217 cells and 18590 genes were identified according to quality control criteria that excluded low-quality cells and genes (2A). Correlation analysis also found that nCount was not correlated with Percent.mt, but was highly correlated with nFeature (2B). The NormalizeData normalization process screened out the top 1500 highly variable genes (2C). The ScaleData function performs Z-score standardization on the data and then carries out PC dimension reduction to select the top 20 PC (2D). The distribution of cells in each sample between PC1 and PC2 is shown in 2E. The tSNE algorithm divided the cells of the sepsis non-survivor group into 13 independent clusters (2F). Subsequently, 13 independent cell clusters were annotated into 7 types of cells through singleR package based on the expression pattern of the marker gene (2G). Clusters of 6, 8, and 10 were annotated as monocytes (2G and H). In addition, the annotation results were also diagnosed, and the results showed that monocytes had a higher score (2I).

Figure S3. Identification of monocyte-related signaling pathways. **A**, Comparison of the overall information flow of each signaling pathway. **B**, The outgoing signals related to monocytes in sepsis survivor and non-survivor groups, respectively. **C**, The incoming signals related to monocytes in sepsis survivor and non-survivor groups, respectively. **D**, Comparison of the outgoing signals related to monocytes in the sepsis survivor and non-survivor groups side by side. **E**, Comparison of the incoming signals related to monocytes in the sepsis survivor and non-survivor groups side by side.

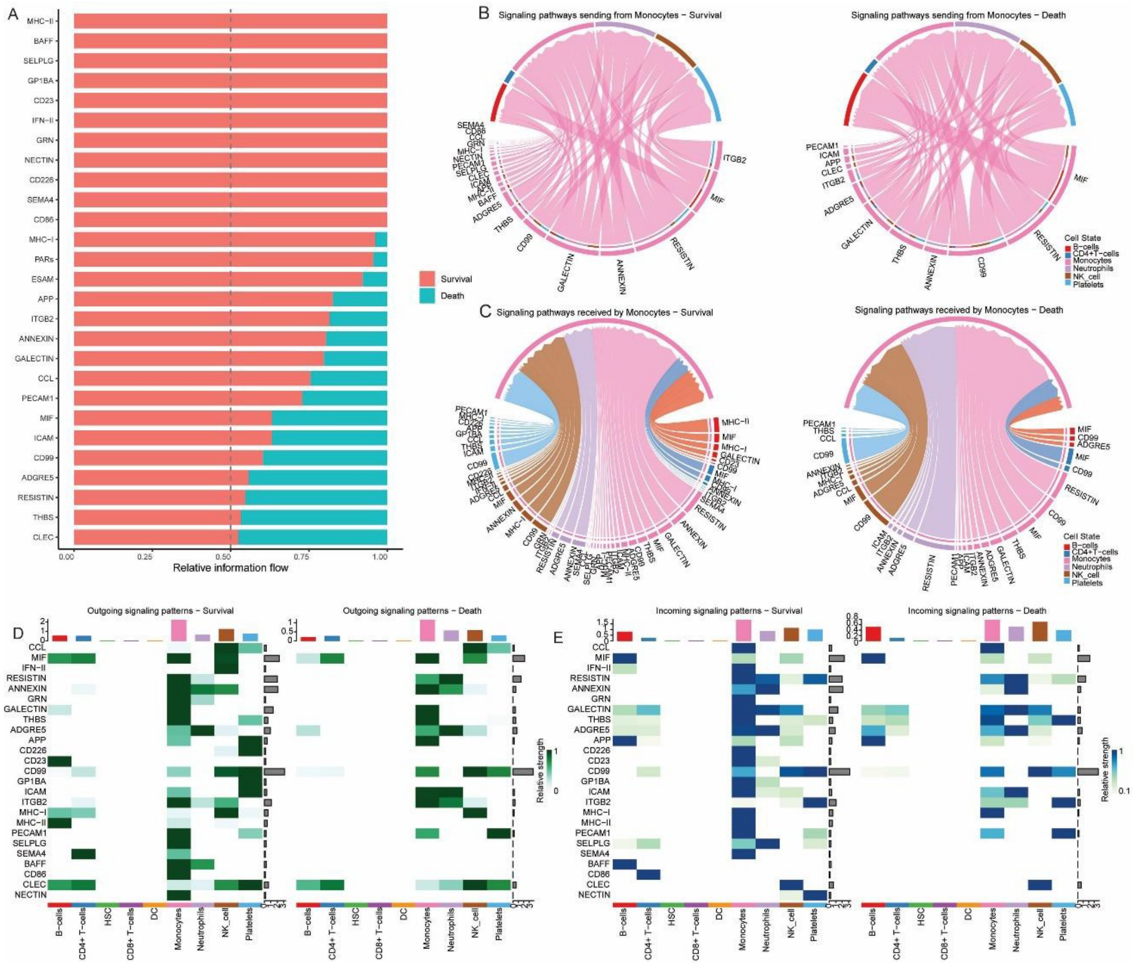

Figure S4. The expression of CD4, CD47, ITGAX, and PLXNB2 in GSE54514 dataset. Data are reported as median and interquartile range. Wilcoxon test. *P<0.05; **P<0.01.

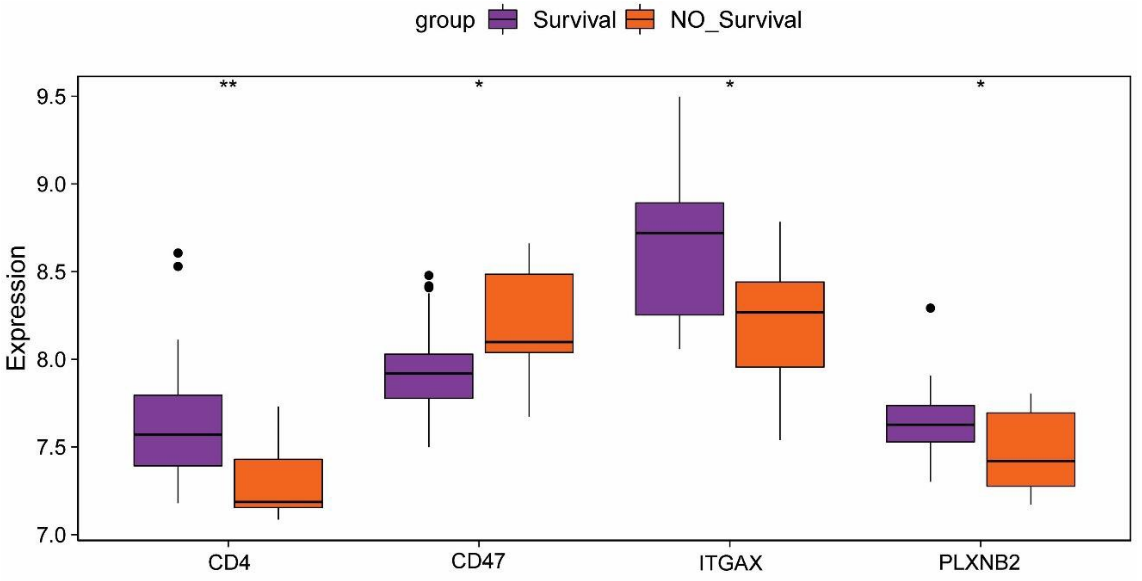

Figure S5. The expression of CCR1, CD4, CD47, ITGAX, LILRB1, and PLXNB2 was verified by real time PCR. Data are reported as the mean±SD. Student's *t*-test.

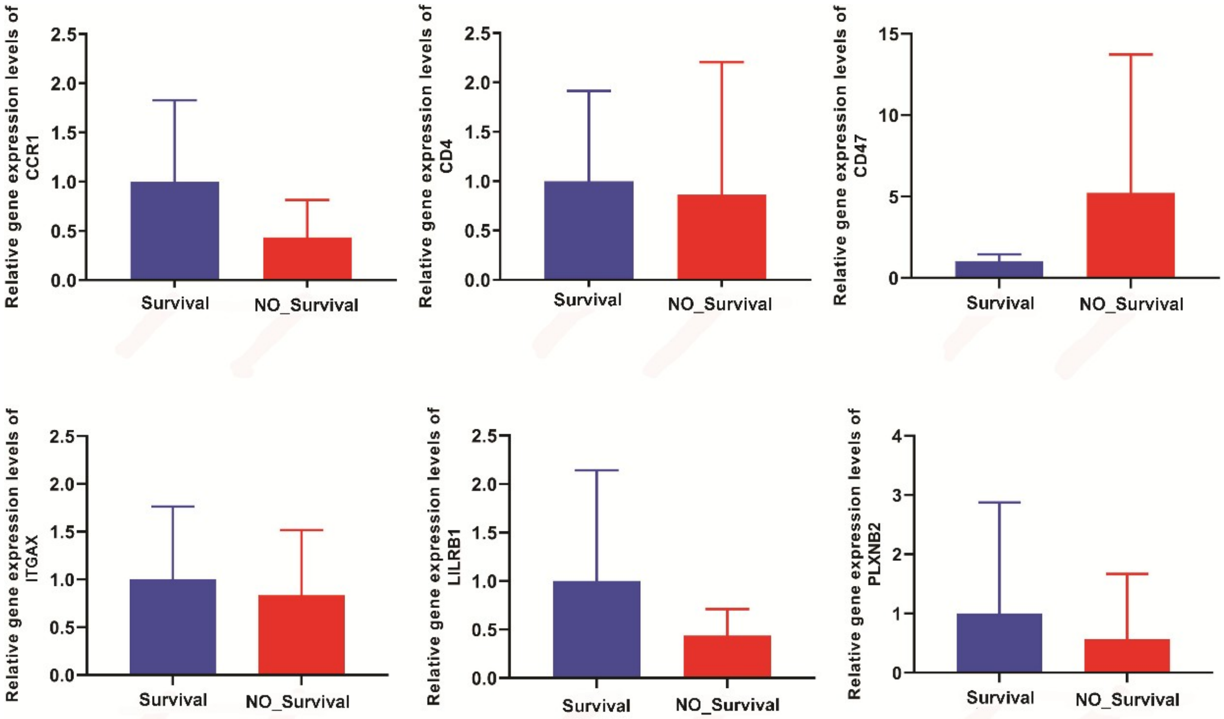

Table S1 Detailed information of the samples in the GSE167363, GSE95233, and GSE54514 datasets.

GSE167363 dataset

!Sample_geo_accession	!Sample_title	!Sample_organism_ch1	!Sample_source_name_ch1
GSM5102900	HC1	Homo sapiens	PBMCs from non-sepsis donor 1.
GSM5102901	HC2	Homo sapiens	PBMCs from non-sepsis donor 2.
GSM5102904	S1 T0	Homo sapiens	PBMCs from the survived septic patient in the Intensive Care Unit (ICU) at 0 hours from sepsis recognition.
GSM5102905	S1 T6	Homo sapiens	PBMCs from the survived septic patient in the Intensive Care Unit (ICU) at 6 hours from sepsis recognition.
GSM5511353	S2_T0	Homo sapiens	PBMCs from the survived septic patient in the Intensive Care Unit (ICU) at 0 hours from sepsis recognition.
GSM5511354	S2_T6	Homo sapiens	PBMCs from the survived septic patient in the Intensive Care Unit (ICU) at 6 hours from sepsis recognition.
GSM5511355	S3_T0	Homo sapiens	PBMCs from the survived septic patient in the Intensive Care Unit (ICU) at 0 hours from sepsis recognition.
GSM5511356	S3_T6	Homo sapiens	PBMCs from the survived septic patient in the Intensive Care Unit (ICU) at 6 hours from sepsis recognition.
GSM5102902	NS LS T0	Homo sapiens	PBMCs from the non-survived septic patient in the Intensive Care Unit (ICU) at 0 hours from sepsis recognition.
GSM5102903	NS LS T6	Homo sapiens	PBMCs from the non-survived septic patient in the Intensive Care Unit (ICU) at 6 hours from sepsis recognition.
GSM5511351	NS ES_T0	Homo sapiens	PBMCs from the non-survived septic patient in the Intensive Care Unit (ICU) at 0 hours from sepsis recognition.
GSM5511352	NS ES_T6	Homo sapiens	PBMCs from the non-survived septic patient in the Intensive Care Unit (ICU) at 6 hours from sepsis recognition.

GSE95233 dataset

!Sample_geo_accession	!Sample_characteristics_ch1	!Sample_source_name_ch1	!Sample_organism_ch1	!Sample_characteristics_ch1	!Sample_characteristics_ch1	!Sample_characteristics_ch1
GSM2500349	survival: NA	Control 500	Homo sapiens	gender: F	age: 52	time point: NA
GSM2500350	survival: NA	Control 526	Homo sapiens	gender: F	age: 53	time point: NA
GSM2500351	survival: NA	Control 529	Homo sapiens	gender: F	age: 52	time point: NA
GSM2500352	survival: NA	Control 530	Homo sapiens	gender: F	age: 53	time point: NA
GSM2500353	survival: NA	Control 535	Homo sapiens	gender: F	age: 76	time point: NA
GSM2500354	survival: NA	Control 553	Homo sapiens	gender: F	age: 50	time point: NA
GSM2500355	survival: NA	Control 598	Homo sapiens	gender: F	age: 57	time point: NA
GSM2500356	survival: NA	Control 628	Homo sapiens	gender: F	age: 61	time point: NA
GSM2500357	survival: NA	Control 742	Homo sapiens	gender: F	age: 51	time point: NA
GSM2500358	survival: NA	Control 789	Homo sapiens	gender: F	age: 52	time point: NA
GSM2500359	survival: NA	Control 793	Homo sapiens	gender: F	age: 67	time point: NA
GSM2500360	survival: NA	Control 511	Homo sapiens	gender: M	age: 61	time point: NA
GSM2500361	survival: NA	Control 513	Homo sapiens	gender: M	age: 57	time point: NA

GSM2500362	survival: NA	Control 517	Homo sapiens	gender: M	age: 60	time point: NA
GSM2500363	survival: NA	Control 523	Homo sapiens	gender: M	age: 57	time point: NA
GSM2500364	survival: NA	Control 528	Homo sapiens	gender: M	age: 67	time point: NA
GSM2500365	survival: NA	Control 530	Homo sapiens	gender: M	age: 56	time point: NA
GSM2500366	survival: NA	Control 532	Homo sapiens	gender: M	age: 62	time point: NA
GSM2500367	survival: NA	Control 534	Homo sapiens	gender: M	age: 52	time point: NA
GSM2500368	survival: NA	Control 535	Homo sapiens	gender: M	age: 57	time point: NA
GSM2500369	survival: NA	Control 537	Homo sapiens	gender: M	age: 60	time point: NA
GSM2500370	survival: NA	Control 538	Homo sapiens	gender: M	age: 59	time point: NA
GSM2500371	survival: Survivor	Patient 108, Day1	Homo sapiens	gender: M	age: 80	time point: D01
GSM2500372	survival: Survivor	Patient 108, Day2	Homo sapiens	gender: M	age: 80	time point: D02
GSM2500373	survival: Survivor	Patient 115, Day1	Homo sapiens	gender: M	age: 59	time point: D01
GSM2500374	survival: Survivor	Patient 115, Day3	Homo sapiens	gender: M	age: 59	time point: D03
GSM2500375	survival: Survivor	Patient 119, Day1	Homo sapiens	gender: F	age: 68	time point: D01
GSM2500376	survival: Survivor	Patient 119, Day2	Homo sapiens	gender: F	age: 68	time point: D02
GSM2500377	survival: Survivor	Patient 121, Day1	Homo sapiens	gender: M	age: 36	time point: D01
GSM2500378	survival: Survivor	Patient 121, Day3	Homo sapiens	gender: M	age: 36	time point: D03
GSM2500379	survival: Survivor	Patient 124, Day1	Homo sapiens	gender: M	age: 66	time point: D01
GSM2500380	survival: Survivor	Patient 124, Day3	Homo sapiens	gender: M	age: 66	time point: D03
GSM2500381	survival: Survivor	Patient 135, Day1	Homo sapiens	gender: M	age: 72	time point: D01
GSM2500382	survival: Survivor	Patient 135, Day3	Homo sapiens	gender: M	age: 72	time point: D03
GSM2500383	survival: Survivor	Patient 142, Day1	Homo sapiens	gender: M	age: 60	time point: D01
GSM2500384	survival: Survivor	Patient 142, Day3	Homo sapiens	gender: M	age: 60	time point: D03
GSM2500385	survival: Survivor	Patient 151, Day1	Homo sapiens	gender: M	age: 81	time point: D01
GSM2500386	survival: Survivor	Patient 151, Day3	Homo sapiens	gender: M	age: 81	time point: D03
GSM2500387	survival: Survivor	Patient 153, Day1	Homo sapiens	gender: F	age: 78	time point: D01
GSM2500388	survival: Survivor	Patient 153, Day3	Homo sapiens	gender: F	age: 78	time point: D03
GSM2500389	survival: Survivor	Patient 16, Day1	Homo sapiens	gender: M	age: 65	time point: D01
GSM2500390	survival: Survivor	Patient 16, Day2	Homo sapiens	gender: M	age: 65	time point: D02
GSM2500391	survival: Survivor	Patient 175, Day1	Homo sapiens	gender: M	age: 37	time point: D01

GSM2500392	survival: Survivor	Patient 175, Day3	Homo sapiens	gender: M	age: 37	time point: D03
GSM2500393	survival: Survivor	Patient 183, Day1	Homo sapiens	gender: M	age: 66	time point: D01
GSM2500394	survival: Survivor	Patient 183, Day2	Homo sapiens	gender: M	age: 66	time point: D02
GSM2500395	survival: Survivor	Patient 19, Day1	Homo sapiens	gender: M	age: 80	time point: D01
GSM2500396	survival: Survivor	Patient 19, Day2	Homo sapiens	gender: M	age: 80	time point: D02
GSM2500397	survival: Survivor	Patient 209, Day1	Homo sapiens	gender: M	age: 83	time point: D01
GSM2500398	survival: Survivor	Patient 209, Day3	Homo sapiens	gender: M	age: 83	time point: D03
GSM2500399	survival: Survivor	Patient 211, Day1	Homo sapiens	gender: F	age: 34	time point: D01
GSM2500400	survival: Survivor	Patient 211, Day3	Homo sapiens	gender: F	age: 34	time point: D03
GSM2500401	survival: Survivor	Patient 213, Day1	Homo sapiens	gender: F	age: 27	time point: D01
GSM2500402	survival: Survivor	Patient 213, Day2	Homo sapiens	gender: F	age: 27	time point: D02
GSM2500403	survival: Survivor	Patient 215, Day1	Homo sapiens	gender: M	age: 55	time point: D01
GSM2500404	survival: Survivor	Patient 215, Day2	Homo sapiens	gender: M	age: 55	time point: D02
GSM2500405	survival: Survivor	Patient 21, Day1	Homo sapiens	gender: M	age: 76	time point: D01
GSM2500406	survival: Survivor	Patient 21, Day3	Homo sapiens	gender: M	age: 76	time point: D03
GSM2500407	survival: Survivor	Patient 248, Day1	Homo sapiens	gender: M	age: 66	time point: D01
GSM2500408	survival: Survivor	Patient 248, Day2	Homo sapiens	gender: M	age: 66	time point: D02
GSM2500409	survival: Survivor	Patient 27, Day1	Homo sapiens	gender: F	age: 42	time point: D01
GSM2500410	survival: Survivor	Patient 27, Day2	Homo sapiens	gender: F	age: 42	time point: D02
GSM2500411	survival: Survivor	Patient 32, Day1	Homo sapiens	gender: F	age: 76	time point: D01
GSM2500412	survival: Survivor	Patient 32, Day3	Homo sapiens	gender: F	age: 76	time point: D03
GSM2500413	survival: Survivor	Patient 33, Day1	Homo sapiens	gender: M	age: 75	time point: D01
GSM2500414	survival: Survivor	Patient 33, Day3	Homo sapiens	gender: M	age: 75	time point: D03
GSM2500415	survival: Survivor	Patient 43, Day1	Homo sapiens	gender: M	age: 54	time point: D01
GSM2500416	survival: Survivor	Patient 43, Day3	Homo sapiens	gender: M	age: 54	time point: D03
GSM2500417	survival: Survivor	Patient 44, Day1	Homo sapiens	gender: M	age: 69	time point: D01
GSM2500418	survival: Survivor	Patient 44, Day3	Homo sapiens	gender: M	age: 69	time point: D03
GSM2500419	survival: Survivor	Patient 46, Day1	Homo sapiens	gender: F	age: 65	time point: D01
GSM2500420	survival: Survivor	Patient 46, Day3	Homo sapiens	gender: F	age: 65	time point: D03
GSM2500421	survival: Survivor	Patient 54, Day1	Homo sapiens	gender: M	age: 63	time point: D01

GSM2500422	survival: Survivor	Patient 54, Day3	Homo sapiens	gender: M	age: 63	time point: D03
GSM2500423	survival: Survivor	Patient 69, Day1	Homo sapiens	gender: M	age: 76	time point: D01
GSM2500424	survival: Survivor	Patient 69, Day3	Homo sapiens	gender: M	age: 76	time point: D03
GSM2500425	survival: Survivor	Patient 70, Day1	Homo sapiens	gender: F	age: 85	time point: D01
GSM2500426	survival: Survivor	Patient 70, Day2	Homo sapiens	gender: F	age: 85	time point: D02
GSM2500427	survival: Survivor	Patient 72, Day1	Homo sapiens	gender: M	age: 25	time point: D01
GSM2500428	survival: Survivor	Patient 72, Day3	Homo sapiens	gender: M	age: 25	time point: D03
GSM2500429	survival: Survivor	Patient 74, Day1	Homo sapiens	gender: M	age: 70	time point: D01
GSM2500430	survival: Survivor	Patient 74, Day2	Homo sapiens	gender: M	age: 70	time point: D02
GSM2500431	survival: Survivor	Patient 80, Day1	Homo sapiens	gender: F	age: 53	time point: D01
GSM2500432	survival: Survivor	Patient 80, Day3	Homo sapiens	gender: F	age: 53	time point: D03
GSM2500433	survival: Survivor	Patient 86, Day1	Homo sapiens	gender: M	age: 70	time point: D01
GSM2500434	survival: Survivor	Patient 86, Day2	Homo sapiens	gender: M	age: 70	time point: D02
GSM2500435	survival: Survivor	Patient 93, Day1	Homo sapiens	gender: M	age: 58	time point: D01
GSM2500436	survival: Survivor	Patient 93, Day3	Homo sapiens	gender: M	age: 58	time point: D03
GSM2500437	survival: Survivor	Patient 95, Day1	Homo sapiens	gender: M	age: 67	time point: D01
GSM2500438	survival: Survivor	Patient 95, Day3	Homo sapiens	gender: M	age: 67	time point: D03
GSM2500439	survival: Non Survivor	Patient 102, Day1	Homo sapiens	gender: M	age: 85	time point: D01
GSM2500440	survival: Non Survivor	Patient 102, Day2	Homo sapiens	gender: M	age: 85	time point: D02
GSM2500441	survival: Non Survivor	Patient 123, Day1	Homo sapiens	gender: F	age: 67	time point: D01
GSM2500442	survival: Non Survivor	Patient 123, Day2	Homo sapiens	gender: F	age: 67	time point: D02
GSM2500443	survival: Non Survivor	Patient 168, Day1	Homo sapiens	gender: M	age: 47	time point: D01
GSM2500444	survival: Non Survivor	Patient 168, Day2	Homo sapiens	gender: M	age: 47	time point: D02
GSM2500445	survival: Non Survivor	Patient 179, Day1	Homo sapiens	gender: M	age: 65	time point: D01
GSM2500446	survival: Non Survivor	Patient 179, Day2	Homo sapiens	gender: M	age: 65	time point: D02
GSM2500447	survival: Non Survivor	Patient 18, Day1	Homo sapiens	gender: H	age: 66	time point: D01
GSM2500448	survival: Non Survivor	Patient 18, Day2	Homo sapiens	gender: H	age: 66	time point: D02
GSM2500449	survival: Non Survivor	Patient 198, Day1	Homo sapiens	gender: F	age: 47	time point: D01
GSM2500450	survival: Non Survivor	Patient 198, Day3	Homo sapiens	gender: F	age: 47	time point: D03
GSM2500451	survival: Non Survivor	Patient 201, Day1	Homo sapiens	gender: F	age: 48	time point: D01

GSM2500452	survival: Non Survivor	Patient 201, Day2	Homo sapiens	gender: F	age: 48	time point: D02
GSM2500453	survival: Non Survivor	Patient 212, Day1	Homo sapiens	gender: F	age: 45	time point: D01
GSM2500454	survival: Non Survivor	Patient 212, Day2	Homo sapiens	gender: F	age: 45	time point: D02
GSM2500455	survival: Non Survivor	Patient 216, Day1	Homo sapiens	gender: M	age: 59	time point: D01
GSM2500456	survival: Non Survivor	Patient 216, Day3	Homo sapiens	gender: M	age: 59	time point: D03
GSM2500457	survival: Non Survivor	Patient 35, Day1	Homo sapiens	gender: M	age: 78	time point: D01
GSM2500458	survival: Non Survivor	Patient 35, Day3	Homo sapiens	gender: M	age: 78	time point: D03
GSM2500459	survival: Non Survivor	Patient 36, Day1	Homo sapiens	gender: F	age: 55	time point: D01
GSM2500460	survival: Non Survivor	Patient 36, Day3	Homo sapiens	gender: F	age: 55	time point: D03
GSM2500461	survival: Non Survivor	Patient 62, Day1	Homo sapiens	gender: F	age: 70	time point: D01
GSM2500462	survival: Non Survivor	Patient 62, Day3	Homo sapiens	gender: F	age: 70	time point: D03
GSM2500463	survival: Non Survivor	Patient 67, Day1	Homo sapiens	gender: H	age: 72	time point: D01
GSM2500464	survival: Non Survivor	Patient 67, Day3	Homo sapiens	gender: H	age: 72	time point: D03
GSM2500465	survival: Non Survivor	Patient 68, Day1	Homo sapiens	gender: M	age: 46	time point: D01
GSM2500466	survival: Non Survivor	Patient 68, Day3	Homo sapiens	gender: M	age: 46	time point: D03
GSM2500467	survival: Non Survivor	Patient 77, Day1	Homo sapiens	gender: F	age: 78	time point: D01
GSM2500468	survival: Non Survivor	Patient 77, Day2	Homo sapiens	gender: F	age: 78	time point: D02
GSM2500469	survival: Non Survivor	Patient 78, Day1	Homo sapiens	gender: F	age: 53	time point: D01
GSM2500470	survival: Non Survivor	Patient 78, Day3	Homo sapiens	gender: F	age: 53	time point: D03
GSM2500471	survival: Non Survivor	Patient 81, Day1	Homo sapiens	gender: F	age: 54	time point: D01
GSM2500472	survival: Non Survivor	Patient 81, Day3	Homo sapiens	gender: F	age: 54	time point: D03

GSE54514 dataset

!Sample_geo_accession	!Sample_characteristics_ch1	!Sample_characteristics_ch1	!Sample_characteristics_ch1	!Sample_characteristics_ch1	!Sample_characteristics_ch1	!Sample_characteristics_ch1
GSM1317896	disease status: healthy	group_day: HC_D1	group_id: HC_1	gender: F	age (years): 42	severity (apacheii): NA
GSM1317897	disease status: healthy	group_day: HC_D1	group_id: HC_2	gender: F	age (years): 40	severity (apacheii): NA
GSM1317898	disease status: healthy	group_day: HC_D1	group_id: HC_3	gender: M	age (years): 66	severity (apacheii): NA
GSM1317899	disease status: healthy	group_day: HC_D1	group_id: HC_4	gender: M	age (years): 24	severity (apacheii): NA
GSM1317900	disease status: healthy	group_day: HC_D1	group_id: HC_5	gender: F	age (years): 70	severity (apacheii): NA
GSM1317901	disease status: healthy	group_day: HC_D1	group_id: HC_6	gender: F	age (years): 45	severity (apacheii): NA
GSM1317902	disease status: healthy	group_day: HC_D1	group_id: HC_7	gender: F	age (years): 60	severity (apacheii): NA

GSM1317903	disease status: healthy	group_day: HC_D1	group_id: HC_8	gender: F	age (years): 36	severity (apacheii): NA
GSM1317904	disease status: healthy	group_day: HC_D1	group_id: HC_9	gender: M	age (years): 47	severity (apacheii): NA
GSM1317905	disease status: healthy	group_day: HC_D1	group_id: HC_10	gender: F	age (years): 45	severity (apacheii): NA
GSM1317906	disease status: healthy	group_day: HC_D1	group_id: HC_11	gender: F	age (years): 58	severity (apacheii): NA
GSM1317907	disease status: healthy	group_day: HC_D1	group_id: HC_12	gender: F	age (years): 62	severity (apacheii): NA
GSM1317908	disease status: healthy	group_day: HC_D1	group_id: HC_13	gender: M	age (years): 24	severity (apacheii): NA
GSM1317909	disease status: healthy	group_day: HC_D1	group_id: HC_14	gender: M	age (years): 24	severity (apacheii): NA
GSM1317910	disease status: healthy	group_day: HC_D1	group_id: HC_15	gender: F	age (years): 25	severity (apacheii): NA
GSM1317911	disease status: healthy	group_day: HC_D1	group_id: HC_16	gender: F	age (years): 24	severity (apacheii): NA
GSM1317912	disease status: healthy	group_day: HC_D1	group_id: HC_17	gender: F	age (years): 27	severity (apacheii): NA
GSM1317913	disease status: healthy	group_day: HC_D1	group_id: HC_18	gender: M	age (years): 54	severity (apacheii): NA
GSM1317914	disease status: sepsis nonsurvivor	group_day: NS_D1	group_id: NS_19	gender: F	age (years): 66	severity (apacheii): 26
GSM1317915	disease status: sepsis nonsurvivor	group_day: NS_D1	group_id: NS_21	gender: F	age (years): 77	severity (apacheii): 26
GSM1317916	disease status: sepsis nonsurvivor	group_day: NS_D1	group_id: NS_22	gender: M	age (years): 80	severity (apacheii): 20
GSM1317917	disease status: sepsis nonsurvivor	group_day: NS_D1	group_id: NS_23	gender: F	age (years): 54	severity (apacheii): 24
GSM1317918	disease status: sepsis nonsurvivor	group_day: NS_D1	group_id: NS_24	gender: M	age (years): 53	severity (apacheii): 28
GSM1317919	disease status: sepsis nonsurvivor	group_day: NS_D1	group_id: NS_25	gender: M	age (years): 64	severity (apacheii): 23
GSM1317920	disease status: sepsis nonsurvivor	group_day: NS_D1	group_id: NS_26	gender: M	age (years): 72	severity (apacheii): 35
GSM1317921	disease status: sepsis nonsurvivor	group_day: NS_D1	group_id: NS_27	gender: F	age (years): 81	severity (apacheii): 19
GSM1317922	disease status: sepsis nonsurvivor	group_day: NS_D1	group_id: NS_28	gender: F	age (years): 80	severity (apacheii): 17
GSM1317923	disease status: sepsis nonsurvivor	group_day: NS_D2	group_id: NS_19	gender: F	age (years): 66	severity (apacheii): 26
GSM1317924	disease status: sepsis nonsurvivor	group_day: NS_D2	group_id: NS_21	gender: F	age (years): 77	severity (apacheii): 26
GSM1317925	disease status: sepsis nonsurvivor	group_day: NS_D2	group_id: NS_22	gender: M	age (years): 80	severity (apacheii): 20
GSM1317926	disease status: sepsis nonsurvivor	group_day: NS_D2	group_id: NS_23	gender: F	age (years): 54	severity (apacheii): 24
GSM1317927	disease status: sepsis nonsurvivor	group_day: NS_D2	group_id: NS_24	gender: M	age (years): 53	severity (apacheii): 28
GSM1317928	disease status: sepsis nonsurvivor	group_day: NS_D2	group_id: NS_26	gender: M	age (years): 72	severity (apacheii): 35
GSM1317929	disease status: sepsis nonsurvivor	group_day: NS_D2	group_id: NS_27	gender: F	age (years): 81	severity (apacheii): 19
GSM1317930	disease status: sepsis nonsurvivor	group_day: NS_D3	group_id: NS_19	gender: F	age (years): 66	severity (apacheii): 26
GSM1317931	disease status: sepsis nonsurvivor	group_day: NS_D3	group_id: NS_21	gender: F	age (years): 77	severity (apacheii): 26
GSM1317932	disease status: sepsis nonsurvivor	group_day: NS_D3	group_id: NS_22	gender: M	age (years): 80	severity (apacheii): 20

GSM1317933	disease status: sepsis nonsurvivor	group_day: NS_D3	group_id: NS_23	gender: F	age (years): 54	severity (apacheii): 24
GSM1317934	disease status: sepsis nonsurvivor	group_day: NS_D3	group_id: NS_24	gender: M	age (years): 53	severity (apacheii): 28
GSM1317935	disease status: sepsis nonsurvivor	group_day: NS_D3	group_id: NS_26	gender: M	age (years): 72	severity (apacheii): 35
GSM1317936	disease status: sepsis nonsurvivor	group_day: NS_D3	group_id: NS_27	gender: F	age (years): 81	severity (apacheii): 19
GSM1317937	disease status: sepsis nonsurvivor	group_day: NS_D4	group_id: NS_19	gender: F	age (years): 66	severity (apacheii): 26
GSM1317938	disease status: sepsis nonsurvivor	group_day: NS_D4	group_id: NS_20	gender: M	age (years): 50	severity (apacheii): 20
GSM1317939	disease status: sepsis nonsurvivor	group_day: NS_D4	group_id: NS_21	gender: F	age (years): 77	severity (apacheii): 26
GSM1317940	disease status: sepsis nonsurvivor	group_day: NS_D4	group_id: NS_23	gender: F	age (years): 54	severity (apacheii): 24
GSM1317941	disease status: sepsis nonsurvivor	group_day: NS_D4	group_id: NS_24	gender: M	age (years): 53	severity (apacheii): 28
GSM1317942	disease status: sepsis nonsurvivor	group_day: NS_D5	group_id: NS_19	gender: F	age (years): 66	severity (apacheii): 26
GSM1317943	disease status: sepsis nonsurvivor	group_day: NS_D5	group_id: NS_23	gender: F	age (years): 54	severity (apacheii): 24
GSM1317944	disease status: sepsis nonsurvivor	group_day: NS_D5	group_id: NS_24	gender: M	age (years): 53	severity (apacheii): 28
GSM1317945	disease status: sepsis survivor	group_day: S_D1	group_id: S_29	gender: F	age (years): 45	severity (apacheii): 10
GSM1317946	disease status: sepsis survivor	group_day: S_D1	group_id: S_30	gender: F	age (years): 81	severity (apacheii): 24
GSM1317947	disease status: sepsis survivor	group_day: S_D1	group_id: S_31	gender: F	age (years): 75	severity (apacheii): 24
GSM1317948	disease status: sepsis survivor	group_day: S_D1	group_id: S_32	gender: M	age (years): 50	severity (apacheii): 20
GSM1317949	disease status: sepsis survivor	group_day: S_D1	group_id: S_33	gender: M	age (years): 59	severity (apacheii): 33
GSM1317950	disease status: sepsis survivor	group_day: S_D1	group_id: S_34	gender: F	age (years): 83	severity (apacheii): 21
GSM1317951	disease status: sepsis survivor	group_day: S_D1	group_id: S_35	gender: M	age (years): 52	severity (apacheii): 16
GSM1317952	disease status: sepsis survivor	group_day: S_D1	group_id: S_36	gender: M	age (years): 58	severity (apacheii): 23
GSM1317953	disease status: sepsis survivor	group_day: S_D1	group_id: S_37	gender: M	age (years): 75	severity (apacheii): 19
GSM1317954	disease status: sepsis survivor	group_day: S_D1	group_id: S_38	gender: M	age (years): 73	severity (apacheii): 10
GSM1317955	disease status: sepsis survivor	group_day: S_D1	group_id: S_39	gender: F	age (years): 70	severity (apacheii): 16
GSM1317956	disease status: sepsis survivor	group_day: S_D1	group_id: S_40	gender: F	age (years): 86	severity (apacheii): 18
GSM1317957	disease status: sepsis survivor	group_day: S_D1	group_id: S_41	gender: F	age (years): 60	severity (apacheii): 17
GSM1317958	disease status: sepsis survivor	group_day: S_D1	group_id: S_42	gender: M	age (years): 43	severity (apacheii): 9
GSM1317959	disease status: sepsis survivor	group_day: S_D1	group_id: S_43	gender: F	age (years): 30	severity (apacheii): 15
GSM1317960	disease status: sepsis survivor	group_day: S_D1	group_id: S_44	gender: F	age (years): 18	severity (apacheii): 11
GSM1317961	disease status: sepsis survivor	group_day: S_D1	group_id: S_45	gender: F	age (years): 68	severity (apacheii): 32
GSM1317962	disease status: sepsis survivor	group_day: S_D1	group_id: S_46	gender: F	age (years): 52	severity (apacheii): 25

GSM1317963	disease status: sepsis survivor	group_day: S_D1	group_id: S_47	gender: M	age (years): 32	severity (apacheii): 20
GSM1317964	disease status: sepsis survivor	group_day: S_D1	group_id: S_48	gender: F	age (years): 67	severity (apacheii): 19
GSM1317965	disease status: sepsis survivor	group_day: S_D1	group_id: S_49	gender: F	age (years): 47	severity (apacheii): 20
GSM1317966	disease status: sepsis survivor	group_day: S_D1	group_id: S_50	gender: F	age (years): 29	severity (apacheii): 13
GSM1317967	disease status: sepsis survivor	group_day: S_D1	group_id: S_51	gender: M	age (years): 65	severity (apacheii): 23
GSM1317968	disease status: sepsis survivor	group_day: S_D1	group_id: S_52	gender: F	age (years): 64	severity (apacheii): 17
GSM1317969	disease status: sepsis survivor	group_day: S_D1	group_id: S_53	gender: F	age (years): 32	severity (apacheii): 12
GSM1317970	disease status: sepsis survivor	group_day: S_D1	group_id: S_54	gender: M	age (years): 60	severity (apacheii): 15
GSM1317971	disease status: sepsis survivor	group_day: S_D2	group_id: S_30	gender: F	age (years): 81	severity (apacheii): 24
GSM1317972	disease status: sepsis survivor	group_day: S_D2	group_id: S_31	gender: F	age (years): 75	severity (apacheii): 24
GSM1317973	disease status: sepsis survivor	group_day: S_D2	group_id: S_32	gender: M	age (years): 50	severity (apacheii): 20
GSM1317974	disease status: sepsis survivor	group_day: S_D2	group_id: S_33	gender: M	age (years): 59	severity (apacheii): 33
GSM1317975	disease status: sepsis survivor	group_day: S_D2	group_id: S_34	gender: F	age (years): 83	severity (apacheii): 21
GSM1317976	disease status: sepsis survivor	group_day: S_D2	group_id: S_35	gender: M	age (years): 52	severity (apacheii): 16
GSM1317977	disease status: sepsis survivor	group_day: S_D2	group_id: S_36	gender: M	age (years): 58	severity (apacheii): 23
GSM1317978	disease status: sepsis survivor	group_day: S_D2	group_id: S_37	gender: M	age (years): 75	severity (apacheii): 19
GSM1317979	disease status: sepsis survivor	group_day: S_D2	group_id: S_38	gender: M	age (years): 73	severity (apacheii): 10
GSM1317980	disease status: sepsis survivor	group_day: S_D2	group_id: S_39	gender: F	age (years): 70	severity (apacheii): 16
GSM1317981	disease status: sepsis survivor	group_day: S_D2	group_id: S_40	gender: F	age (years): 86	severity (apacheii): 18
GSM1317982	disease status: sepsis survivor	group_day: S_D2	group_id: S_41	gender: F	age (years): 60	severity (apacheii): 17
GSM1317983	disease status: sepsis survivor	group_day: S_D2	group_id: S_42	gender: M	age (years): 43	severity (apacheii): 9
GSM1317984	disease status: sepsis survivor	group_day: S_D2	group_id: S_43	gender: F	age (years): 30	severity (apacheii): 15
GSM1317985	disease status: sepsis survivor	group_day: S_D2	group_id: S_45	gender: F	age (years): 68	severity (apacheii): 32
GSM1317986	disease status: sepsis survivor	group_day: S_D2	group_id: S_46	gender: F	age (years): 52	severity (apacheii): 25
GSM1317987	disease status: sepsis survivor	group_day: S_D2	group_id: S_47	gender: M	age (years): 32	severity (apacheii): 20
GSM1317988	disease status: sepsis survivor	group_day: S_D2	group_id: S_48	gender: F	age (years): 67	severity (apacheii): 19
GSM1317989	disease status: sepsis survivor	group_day: S_D2	group_id: S_49	gender: F	age (years): 47	severity (apacheii): 20
GSM1317990	disease status: sepsis survivor	group_day: S_D2	group_id: S_50	gender: F	age (years): 29	severity (apacheii): 13
GSM1317991	disease status: sepsis survivor	group_day: S_D2	group_id: S_51	gender: M	age (years): 65	severity (apacheii): 23
GSM1317992	disease status: sepsis survivor	group_day: S_D2	group_id: S_52	gender: F	age (years): 64	severity (apacheii): 17

GSM1317993	disease status: sepsis survivor	group_day: S_D2	group_id: S_53	gender: F	age (years): 32	severity (apacheii): 12
GSM1317994	disease status: sepsis survivor	group_day: S_D2	group_id: S_54	gender: M	age (years): 60	severity (apacheii): 15
GSM1317995	disease status: sepsis survivor	group_day: S_D3	group_id: S_30	gender: F	age (years): 81	severity (apacheii): 24
GSM1317996	disease status: sepsis survivor	group_day: S_D3	group_id: S_31	gender: F	age (years): 75	severity (apacheii): 24
GSM1317997	disease status: sepsis survivor	group_day: S_D3	group_id: S_32	gender: M	age (years): 50	severity (apacheii): 20
GSM1317998	disease status: sepsis survivor	group_day: S_D3	group_id: S_33	gender: M	age (years): 59	severity (apacheii): 33
GSM1317999	disease status: sepsis survivor	group_day: S_D3	group_id: S_34	gender: F	age (years): 83	severity (apacheii): 21
GSM1318000	disease status: sepsis survivor	group_day: S_D3	group_id: S_35	gender: M	age (years): 52	severity (apacheii): 16
GSM1318001	disease status: sepsis survivor	group_day: S_D3	group_id: S_36	gender: M	age (years): 58	severity (apacheii): 23
GSM1318002	disease status: sepsis survivor	group_day: S_D3	group_id: S_38	gender: M	age (years): 73	severity (apacheii): 10
GSM1318003	disease status: sepsis survivor	group_day: S_D3	group_id: S_39	gender: F	age (years): 70	severity (apacheii): 16
GSM1318004	disease status: sepsis survivor	group_day: S_D3	group_id: S_42	gender: M	age (years): 43	severity (apacheii): 9
GSM1318005	disease status: sepsis survivor	group_day: S_D3	group_id: S_43	gender: F	age (years): 30	severity (apacheii): 15
GSM1318006	disease status: sepsis survivor	group_day: S_D3	group_id: S_45	gender: F	age (years): 68	severity (apacheii): 32
GSM1318007	disease status: sepsis survivor	group_day: S_D3	group_id: S_46	gender: F	age (years): 52	severity (apacheii): 25
GSM1318008	disease status: sepsis survivor	group_day: S_D3	group_id: S_47	gender: M	age (years): 32	severity (apacheii): 20
GSM1318009	disease status: sepsis survivor	group_day: S_D3	group_id: S_48	gender: F	age (years): 67	severity (apacheii): 19
GSM1318010	disease status: sepsis survivor	group_day: S_D3	group_id: S_49	gender: F	age (years): 47	severity (apacheii): 20
GSM1318011	disease status: sepsis survivor	group_day: S_D3	group_id: S_50	gender: F	age (years): 29	severity (apacheii): 13
GSM1318012	disease status: sepsis survivor	group_day: S_D3	group_id: S_51	gender: M	age (years): 65	severity (apacheii): 23
GSM1318013	disease status: sepsis survivor	group_day: S_D3	group_id: S_52	gender: F	age (years): 64	severity (apacheii): 17
GSM1318014	disease status: sepsis survivor	group_day: S_D3	group_id: S_53	gender: F	age (years): 32	severity (apacheii): 12
GSM1318015	disease status: sepsis survivor	group_day: S_D3	group_id: S_54	gender: M	age (years): 60	severity (apacheii): 15
GSM1318016	disease status: sepsis survivor	group_day: S_D4	group_id: S_30	gender: F	age (years): 81	severity (apacheii): 24
GSM1318017	disease status: sepsis survivor	group_day: S_D4	group_id: S_33	gender: M	age (years): 59	severity (apacheii): 33
GSM1318018	disease status: sepsis survivor	group_day: S_D4	group_id: S_35	gender: M	age (years): 52	severity (apacheii): 16
GSM1318019	disease status: sepsis survivor	group_day: S_D4	group_id: S_39	gender: F	age (years): 70	severity (apacheii): 16
GSM1318020	disease status: sepsis survivor	group_day: S_D4	group_id: S_41	gender: F	age (years): 60	severity (apacheii): 17
GSM1318021	disease status: sepsis survivor	group_day: S_D4	group_id: S_42	gender: M	age (years): 43	severity (apacheii): 9
GSM1318022	disease status: sepsis survivor	group_day: S_D4	group_id: S_43	gender: F	age (years): 30	severity (apacheii): 15

GSM1318023	disease status: sepsis survivor	group_day: S_D4	group_id: S_45	gender: F	age (years): 68	severity (apacheii): 32
GSM1318024	disease status: sepsis survivor	group_day: S_D4	group_id: S_46	gender: F	age (years): 52	severity (apacheii): 25
GSM1318025	disease status: sepsis survivor	group_day: S_D4	group_id: S_48	gender: F	age (years): 67	severity (apacheii): 19
GSM1318026	disease status: sepsis survivor	group_day: S_D4	group_id: S_49	gender: F	age (years): 47	severity (apacheii): 20
GSM1318027	disease status: sepsis survivor	group_day: S_D4	group_id: S_50	gender: F	age (years): 29	severity (apacheii): 13
GSM1318028	disease status: sepsis survivor	group_day: S_D4	group_id: S_51	gender: M	age (years): 65	severity (apacheii): 23
GSM1318029	disease status: sepsis survivor	group_day: S_D4	group_id: S_54	gender: M	age (years): 60	severity (apacheii): 15
GSM1318030	disease status: sepsis survivor	group_day: S_D5	group_id: S_30	gender: F	age (years): 81	severity (apacheii): 24
GSM1318031	disease status: sepsis survivor	group_day: S_D5	group_id: S_33	gender: M	age (years): 59	severity (apacheii): 33
GSM1318032	disease status: sepsis survivor	group_day: S_D5	group_id: S_35	gender: M	age (years): 52	severity (apacheii): 16
GSM1318033	disease status: sepsis survivor	group_day: S_D5	group_id: S_42	gender: M	age (years): 43	severity (apacheii): 9
GSM1318034	disease status: sepsis survivor	group_day: S_D5	group_id: S_43	gender: F	age (years): 30	severity (apacheii): 15
GSM1318035	disease status: sepsis survivor	group_day: S_D5	group_id: S_45	gender: F	age (years): 68	severity (apacheii): 32
GSM1318036	disease status: sepsis survivor	group_day: S_D5	group_id: S_46	gender: F	age (years): 52	severity (apacheii): 25
GSM1318037	disease status: sepsis survivor	group_day: S_D5	group_id: S_49	gender: F	age (years): 47	severity (apacheii): 20
GSM1318038	disease status: sepsis survivor	group_day: S_D5	group_id: S_50	gender: F	age (years): 29	severity (apacheii): 13
GSM1318039	disease status: sepsis survivor	group_day: S_D5	group_id: S_51	gender: M	age (years): 65	severity (apacheii): 23
GSM1318040	disease status: sepsis survivor	group_day: S_D5	group_id: S_54	gender: M	age (years): 60	severity (apacheii): 15
GSM1318041	disease status: healthy	group_day: HC_D5	group_id: HC_1	gender: F	age (years): 42	severity (apacheii): NA
GSM1318042	disease status: healthy	group_day: HC_D5	group_id: HC_2	gender: F	age (years): 40	severity (apacheii): NA
GSM1318043	disease status: healthy	group_day: HC_D5	group_id: HC_3	gender: M	age (years): 66	severity (apacheii): NA
GSM1318044	disease status: healthy	group_day: HC_D5	group_id: HC_4	gender: M	age (years): 24	severity (apacheii): NA
GSM1318045	disease status: healthy	group_day: HC_D5	group_id: HC_5	gender: F	age (years): 70	severity (apacheii): NA
GSM1318046	disease status: healthy	group_day: HC_D5	group_id: HC_6	gender: F	age (years): 45	severity (apacheii): NA
GSM1318047	disease status: healthy	group_day: HC_D5	group_id: HC_7	gender: F	age (years): 60	severity (apacheii): NA
GSM1318048	disease status: healthy	group_day: HC_D5	group_id: HC_8	gender: F	age (years): 36	severity (apacheii): NA
GSM1318049	disease status: healthy	group_day: HC_D5	group_id: HC_9	gender: M	age (years): 47	severity (apacheii): NA
GSM1318050	disease status: healthy	group_day: HC_D5	group_id: HC_10	gender: F	age (years): 45	severity (apacheii): NA
GSM1318051	disease status: healthy	group_day: HC_D5	group_id: HC_11	gender: F	age (years): 58	severity (apacheii): NA
GSM1318052	disease status: healthy	group_day: HC_D5	group_id: HC_12	gender: F	age (years): 62	severity (apacheii): NA

GSM1318053	disease status: healthy	group_day: HC_D5	group_id: HC_13	gender: M	age (years): 24	severity (apacheii): NA
GSM1318054	disease status: healthy	group_day: HC_D5	group_id: HC_14	gender: M	age (years): 24	severity (apacheii): NA
GSM1318055	disease status: healthy	group_day: HC_D5	group_id: HC_15	gender: F	age (years): 25	severity (apacheii): NA
GSM1318056	disease status: healthy	group_day: HC_D5	group_id: HC_16	gender: F	age (years): 24	severity (apacheii): NA
GSM1318057	disease status: healthy	group_day: HC_D5	group_id: HC_17	gender: F	age (years): 27	severity (apacheii): NA
GSM1318058	disease status: healthy	group_day: HC_D5	group_id: HC_18	gender: M	age (years): 54	severity (apacheii): NA

Table S2. Clinical information of samples in the GSE65682 dataset.

	ALL n=479	Non-survivor n=114	Survivor n=365	P overall
Gender				0.549
Female	207 (43.2%)	46 (40.4%)	161 (44.1%)	
Male	272 (56.8%)	68 (59.6%)	204 (55.9%)	
Age				0.159
≤60	193 (40.3%)	39 (34.2%)	154 (42.2%)	
>60	286 (59.7%)	75 (65.8%)	211 (57.8%)	
Pneumonia diagnosis				1.000
CAP	106 (57.9%)	23 (57.5%)	83 (58.0%)	
HAP	77 (42.1%)	17 (42.5%)	60 (42.0%)	
Diabetes mellitus				0.783
Yes	89 (22.8%)	22 (24.4%)	67 (22.3%)	
No	301 (77.2%)	68 (75.6%)	233 (77.7%)	

CAP: community-acquired pneumonia; HAP: hospital-acquired pneumonia. Chi-squared test.

Table S3. The interaction score among hub genes.

Node1	Node2	Interaction score
CCR1	CD4	0.838
CCR1	ITGAX	0.629
CD4	CD47	0.548
CD4	LILRB1	0.649
CD4	ITGAX	0.952
CD47	ITGAX	0.466
ITGAX	LILRB1	0.466
